# The effect of SSRIs on unconditioned anxiety: a systematic review and meta-analysis of animal studies

**DOI:** 10.1007/s00213-024-06645-2

**Published:** 2024-07-09

**Authors:** Elise J. Heesbeen, Tatum van Kampen, P. Monika Verdouw, Caspar van Lissa, Elisabeth Y. Bijlsma, Lucianne Groenink

**Affiliations:** 1 Division of Pharmacology, Utrecht Institute for Pharmaceutical Sciences, Utrecht University, Utrecht, The Netherlands; 2 Department of Methodology, Tilburg University, Tilburg, The Netherlands

**Keywords:** Anxiety, Unconditioned anxiety, SSRIs, Elevated plus maze, Marble burying, Ultrasonic vocalization, Stress-induced hyperthermia, Systematic review, Meta-analysis, 5-HT reuptake inhibitor

## Abstract

**Rationale:**

Selective serotonin reuptake inhibitors (SSRIs) are the first choice of treatment for anxiety-like disorders. However, which aspects of anxiety are affected by SSRIs is not yet fully understood.

**Objective:**

We aimed to systematically review the effect of six clinically effective SSRIs on four aspects of unconditioned anxiety: approach-avoidance behaviour (elevated plus maze), repetitive behaviour (marble burying), distress behaviour (ultrasonic vocalization), and activation of the autonomous nervous system (stress-induced hyperthermia).

**Methods:**

We identified publications by searching Medline and Embase databases and assessed the risk of bias. A random effects meta-analysis was performed and moderator effects were analysed with Bayesian penalized meta-regression.

**Results:**

Our search yielded 105 elevated plus maze, 63 marble burying, 11 ultrasonic vocalization, and 7 stress-induced hyperthermia articles. Meta-analysis suggested that SSRIs reduce anxiety-like behaviour in the elevated plus maze, marble burying and ultrasonic vocalization test and that effects are moderated by pre-existing stress conditions (elevated plus maze) and dose dependency (marble burying) but not by duration of treatment or type of SSRI. The reporting quality was low, publication bias was likely, and heterogeneity was high.

**Conclusion:**

SSRIs seem to reduce a broad range of unconditioned anxiety-associated behaviours. These results should be interpreted with caution due to a high risk of bias, likely occurrence of publication bias, substantial heterogeneity and limited moderator data availability. Our review demonstrates the importance of including bias assessments when interpreting meta-analysis results. We further recommend improving the reporting quality, the conduct of animal research, and the publication of all results regardless of significance.

**Supplementary Information:**

The online version contains supplementary material available at 10.1007/s00213-024-06645-2.

## Introduction

Serotonin is known to play an important role in the development and treatment of anxiety-like disorders. These include various anxiety disorders but also obsessive-compulsive disorders and trauma- and stressor-related disorders and they are all paired with a feeling of intense distress. Selective serotonin reuptake inhibitors (SSRIs) are currently the first choice of pharmacological treatment for patients with anxiety-like disorders. However, the exact pathophysiology of anxiety-like disorders and the specific aspects of anxiety that are modulated by these SSRIs are not yet fully understood. Human studies suggest that SSRIs can improve fear extinction (Bui et al. 2013). Also, SSRI treatment in young people could increase the ability to regulate emotions, reduce anger processing, increase positive bias and reduce avoidance of social situations (Murphy et al. 2021). Better insight into how SSRIs specifically affect anxiety-like symptoms could be obtained by performing animal tests. By identifying the anxiety aspects that are affected by SSRIs, we will gain a better understanding of the role serotonin plays in approach-avoidance behaviour, repetitive behaviour, distress behaviour, and activation of the autonomous nervous system. This is interesting for multiple reasons, all relating to the possible identification of new therapeutic targets for patients with anxiety-like disorders. First of all, not all patients experience the therapeutic beneficial effects of SSRIs and therefore there is a need for alternative pharmacological treatments. Identification of anxiety tests in animals that do not show an anxiolytic effect of SSRIs provides an opportunity to investigate whether these tests reflect the SSRI non-responder patient group. Second, SSRIs are known for their delayed therapeutical onset in the treatment of anxiety-like disorders (Lenze et al. 2005; Rickels et al. 2003). Thus, patients do not experience an immediate relief of symptoms after starting treatment and sometimes will even experience a worsening of symptoms for a short period (Offidani et al. 2013). It is, therefore, interesting to investigate whether there are pharmacological treatments that show an acute anxiolytic response in SSRI-sensitive behavioural tests.

Here we build on our previous work in which we systematically reviewed the effect of six clinically-approved SSRIs on tests of conditioned anxiety. The results of this systematic review suggested that the clinical efficacy of SSRIs may be linked to their effects on contextual fear expression and extinction of cued fear (Heesbeen et al. 2023). However, these results did not exclude the possibility that SSRIs also generally inhibit fear-related emotions. A similar data synthesis of the effect of SSRIs on tests of unconditioned anxiety has not yet been carried out. Therefore, with this systematic review, we aimed to establish which aspects of unconditioned anxiety are sensitive to the six clinically-approved SSRIs. We set out to investigate the effect of SSRIs on four aspects of unconditioned anxiety: approach-avoidance behaviour, repetitive behaviour, distress behaviour, and activation of the autonomous nervous system. These aspects are tested in the elevated plus maze, marble burying test, ultrasonic vocalization test, and stress-induced hyperthermia test, respectively.

The initial animal tests that were developed to investigate anxiolytic drug effects were based on the experimentally induced conflict between approach and avoidance behaviour. The elevated plus maze is based on this principle and is most commonly used for anxiolytic drug screening (Ravi and Eerike 2022), and was therefore selected for this review instead of other approach-avoidance tests such as the light-dark box. Rodents experience a conflict between exploring the open arms (approach) and fearing the open arms and thus favouring the enclosed arms (avoidance) (Pellow et al. 1985). This conflict of approach and avoidance is an important concept of human anxiety and individuals with anxiety-like disorders typically show increased avoidance behaviour. Another way to assess anxiolytic drug efficacy is by performing the ultrasonic vocalization test. This test is based on the principle that aversive events (e.g. separation from littermates, electric shock, mild restraint stress) in neonatal or adult rodents induce distress behaviour which manifests in ultrasonic vocalizations (USVs) (Cuomo et al. 1988); Gardner 1985; Winslow and Insel 1991a; b). The number of emitted USVs indicates the stress that is experienced by individuals with anxiety after exposure to unavoidable aversive situations (Jelen et al. 2003). Anxiolytic drug efficacy can also be assessed in the stress-induced hyperthermia test, which assesses the increase in body temperature after exposure to a stressor, as a proxy of activation of the autonomic nervous system. Since stress leads to consistent body temperature changes in humans, body temperature as measured in the stress-induced hyperthermia test remains a good physiological readout of a stress response (Vinkers et al. 2013). In addition to the aforementioned anxiety aspects, it is also possible to measure the persistent and repetitive behaviour of animals with the marble burying test which is based on the naturally occurring tendency of rodents to bury objects situated in their environment (De Boer and Koolhaas 2003; Poling et al. 1981). This behaviour is thought to be more ethologically relevant to obsessive-compulsive disorder than to other anxiety-like disorders since the repetitive behaviour resembles the stereotype pattern behaviour that is often observed in obsessive-compulsive patients (Thomas et al. 2009; Witkin 2008).

In addition to reviewing the effects of SSRIs in these four tests, we performed moderator analyses to determine whether the effects of SSRIs were dependent on the type of SSRI under study and the duration of treatment, the presence of a pre-existing anxiety condition, sex differences and the species under study. The results of the moderator analysis could help to optimize the experimental set-up for studying unconditioned anxiety.

## Materials and methods

This systematic review was written according to the guidelines as described in the Preferred Reporting Items for Systematic Reviews and Meta-Analysis (PRISMA) statement (Page et al. 2021).

### Study protocol

This systematic review was performed according to a preregistered protocol (PROSPERO, CRD42022371871) which was registered on 16 December 2022 and can be accessed through this website: https://www.crd.york.ac.uk/prospero/.

### Literature search and selection

#### Search strategy

A literature search was performed in two large medical databases: Medline and Embase. The search contained all articles published up until the 12th of September 2022. The search strategy was based on the following three components: (1) anxiety test (elevated plus maze or marble burying or ultrasonic vocalization or stress-induced hyperthermia); (2) clinically effective SSRIs (citalopram, escitalopram, fluoxetine, fluvoxamine, paroxetine, and sertraline); and (3) all animals (animal filters from Hooijmans et al. (2010) and de Vries et al. (2014)). We conducted four separate searches, one for each anxiety test. The specific search string for the animal test was combined with the second and third search components. The complete search string can be found in Supplementary file S1. The four sets of articles that were obtained through this search were imported and deduplicated in Endnote. The web program Rayyan (https://www.rayyan.ai/) was subsequently used to perform the study selection on the remaining articles.

#### Study selection

All articles obtained through the literature search were screened independently by two reviewers (TvK and EH) according to the inclusion (Table 1) and exclusion criteria (Table 2) as described in the preregistered protocol. Two screening phases took place: (1) screening of the titles and abstracts of all the unique articles; (2) screening of the full text of possibly relevant articles to determine eligibility. Any discrepancies that occurred in the screening phases were resolved by a discussion between the two reviewers, a third reviewer (EYB or LG) was only consulted whenever the conflict could not be resolved.

**Table 1:** Inclusion criteria

Category	Inclusion criteria
Type of study	Originally peer-reviewed published studies
	Placebo or vehicle-controlled studies
Type of animals	All non-human animals
Type of intervention	One of six clinically effective SSRIs
	All dosing schedules
Outcome measures	Any outcome parameter assessing the level of anxiety-like behaviour in the respective anxiety test
Language	English

**Table 2:** Exclusion criteria, sorted by priority. Criteria 1–5 determined eligibility in the first phase (title and abstract screening), and criteria 1–15 determined eligibility in the second phase (full text screening)

	Exclusion criteria
1	Not an original full publication
2	Not an in vivo animal study, but a human, in vitro or ex vivo study
3	No use of elevated plus maze, marble burying, ultrasonic vocalization or stress-induced hyperthermia test, or test not used to measure anxiety
4	No SSRI treatment used
5	SSRI not administered directly to subject but through maternal exposure
6	No appropriate placebo or vehicle-controlled experiment
7	No information available/retrievable within one reference on the protocol of the anxiety test
8	No information available/retrievable on specific SSRI used
9	No results reported from the used anxiety test
10	Use of an additional pharmacological treatment before/during/after SSRI treatment and before the anxiety test
11	SSRI treatment not tested on one of the four defined anxiety tests
12	Studies on one of the four defined anxiety tests where the SSRI was not given up until or within 24 h of measuring anxiety behaviour
13	Animals are tested in other behavioural tests before tested in the anxiety test of interest
14	Pretesting of the anxiety test is not part of selection within the test procedure
15	Full article text not retrievable or not in English language

### Extraction of study characteristics

The general and test-specific characteristics as summarized in Table 3 were extracted from the included articles by one reviewer (TvK) and checked by the second reviewer (EH).

**Table 3:** General and test-specific characteristics and the extracted outcome parameters from the included articles

General characteristics	
Experimental design	Housing
	Time of testing
	Day/night schedule
Animal model	Species
	Strain
	Age/body weight at time of testing
	Sex
	Disease induction
Intervention	Type of SSRI
	Doses
	Duration of treatment (acute (1x in 24 h)/subchronic (< 7 days)/chronic treatment (> 7 days), for subchronic and chronic the number of days were specified)
	Timing of administration relative to disease induction
Locomotor activity	Change in locomotion (</>/=), locomotion test, locomotion tested during the unconditioned anxiety test (yes/no)
Test specific characteristics	
Marble burying	Total number of marbles
Ultrasonic vocalization	Separation-induced, shock-induced or stress-induced test
	Foot-shock delivery protocol (when applicable)
Stress-induced hyperthermia	Group or individual test
	Time interval between first and second temperature measurement
Test specific outcome parameters	
Elevated plus maze	Time or number of entries in open arm
Marble burying	Number of marbles buried
Ultrasonic vocalization	Number of vocalizations or duration of vocalizations
Stress-induced hyperthermia	Baseline body temperature
	Mean difference in body temperature after SSRI treatment

### Risk of bias assessment

SYRCLE’s risk of bias tool was used to assess the study quality of all included articles (Hooijmans et al. 2014). This tool uses the following three ratings: high, unclear, or low risk of bias. The risk of bias was scored as “low” whenever an article took proper measures to minimise or prevent the risk of bias. However, articles that did not take sufficient measures to limit the risk of bias were scored as “high” (e.g., researchers were not blinded for the experimental conditions). If data was insufficient to evaluate the risk of bias, the respective items of the risk of bias tool were scored as “unclear”. In this tool, six general categories are distinguished to assess the risk of bias, namely: selection bias, performance bias, detection bias, attrition bias, reporting bias, and other biases. Within the latter category, the presence of a conflict of interest was assessed for every article. Two steps were taken to assess “selective outcome reporting” (reporting bias): (1) checking whether the outcome measures mentioned in the method section were also reported in the result section and (2) determining whether the preclinical studies were preregistered by examining the following two databases: Animal Study Registry (https://www.animalstudyregistry.org) and Preclinicaltrials (https://preclinicaltrials.eu/database). We added two additional reporting items to the risk of bias tool to assess whether any randomization and blinding occurred within the experiment. Two reviewers (TvK and EH) independently assessed the risk of bias and discrepancies were resolved by discussion.

### Extraction of outcome data

One reviewer (TvK) extracted the outcome data of the selected articles which was then checked by a second reviewer (EH). The specific outcome measures that were extracted differed per test: (percentage of) time and number of entries in open arm (elevated plus maze); number of marbles buried (marble burying); number and duration of ultrasonic vocalizations (ultrasonic vocalization); and baseline body temperature and difference in body temperature (stress-induced hyperthermia). For each anxiety test, only one outcome measure was extracted per animal to prevent dependency in our data set. The mean, standard deviation (SD) or standard error of the mean (SEM) and number of animals were extracted for both the SSRI and the control group. If the data that needed to be extracted was presented in a different unit than a mean with an SD/SEM (e.g., interquartile range or median), the data was converted by using the appropriate formulas under the assumption that the data was normally distributed. Data was extracted from text, tables, and figures. The latter data was extracted with a digital ruler. If the number of animals was presented as a range, the largest value was used to calculate the corresponding SD. When an article had missing or unclear data, the authors were contacted. If the authors in question could not provide the requested data, the article was removed from the meta-analysis.

Several different outcome measures were reported for the elevated plus maze within the selected articles. The order of importance of these outcome measures was determined based on the predefined estimated relevance of the observed anxiety-like behaviour. The outcomes were ranked as follows: (1) entries in open arms as a percentage of total entries (%EOA), (2) time spent in open arms as a percentage of total time spent in any of the arms (%TOA), (3) absolute number of entries in open arms (EOA) and (4) absolute amount of time spent in open arms (TOA). Articles not reporting any of these four outcome measures were individually evaluated to include the most relevant outcome measure in the analysis.

For the ultrasonic vocalization test, two types of outcome measures were reported by the selected articles: duration and number of vocalizations. The latter outcome measure was determined to be most relevant and thus if an article reported both outcome measures the number of vocalizations was extracted.

We also extracted the effect of SSRIs on locomotor activity from studies that used the elevated plus maze, marble burying and ultrasonic vocalization test. Locomotor activity was extracted from either the anxiety test, a locomotor test that was performed after the anxiety test of interest or a locomotor test that was performed on a separate group of animals. Since these tests rely on physical motor activity, drug effects on locomotor activity could influence the anxiety outcome measure. Descriptive outcome data was extracted as a significant increase/no effect/significant decrease in locomotor activity compared to the placebo-controlled group.

### Data-analysis

Meta-analysis was performed in R (R Core Team 2023) using the R-packages metafor (Viechtbauer 2010) and pema (Van Lissa et al. 2023). The analysis code was preregistered to ensure reproducibility and transparency and can be found via: https://github.com/cjvanlissa/meta_anx_ssri.git. A random effects model was applied since variation in true effect size was expected. Each data set was analysed separately. Effect sizes were obtained through a three-level meta-analysis to account for dependent effect sizes within studies (e.g. due to multiple uses of the control group in dose-response studies) and presented as Hedges’ g with a 95% Confidence Interval (CI) (Van den Noortgate et al. 2015). If articles reported a sample size range, the lowest value was used to calculate the corresponding effect size.

Six categorical predefined moderators were coded to account for the between-studies heterogeneity: type of SSRI, duration of treatment, disease induction, species, sex, and use of pretest. Disease induction is defined as heightened anxiety-related behaviour of subjects due to either pharmacological, genetic or environmental manipulation. A pretest is a selection procedure for the anxiety test of interest to ensure that the animals included in the experiment are sensitive to the investigated anxiety test. For the ultrasonic vocalization and stress-induced hyperthermia, we defined an extra moderator, type of test, which was separation-induced and physical stress-induced for the ultrasonic vocalization and group or individual for the stress-induced hyperthermia. A total of 27 dummy variables were created to define all conditions within these categorical moderators (Table 4). A classical meta-regression model was not identified since the number of moderators was almost identical to the number of available effect sizes (per anxiety test). Therefore, Bayesian regularized meta-regression (BRMA), implemented in the pema package, was used (Van Lissa et al. 2023). BRMA performs moderator selection by shrinking small regression coefficients towards zero with the use of a horseshoe prior, which also aids model identification (Van Lissa et al. 2023). In addition to the categorical moderators, one continuous moderator was included in the analysis: the human equivalent dose (HED). The HED was calculated from the reported dose by using species-specific conversion factors which are based on body surface area (Center for Drug Evaluation and Research 2005, available at: http://www.fda.gov/downloads/Drugs/Guidance/UCM078932.pdf).

**Table 4:** Dummy variables for every categorical moderator

Categorical moderator	Dummy variables
Type of SSRI	Citalopram
	Escitalopram
	Fluoxetine
	Fluvoxamine
	Paroxetine
	Sertraline
Duration of treatment	Acute
	Subchronic
	Chronic
Disease induction	Healthy
	Stress
	Other
Species	Rat
	Mouse
	Gerbil
	Frog
	Hamster
Sex	Male
	Female
	Both
	NR
Use of pretest	Yes
	No
Type of USV	Separation-induced
	Physical stress-induced
Type of SIH	Individual
	Group

The BRMA requires the selection of reference categories to examine the effect of the categorical variables. Dummy variables encode the difference between each remaining category and this reference category. When examining the results, the intercept represents the expected effect size for a study that falls within the reference category for all categorical variables. The effect of dummy variables represents the difference between that category with the reference category. If a dummy variable has a significant effect, that means that that group’s mean differs significantly from the reference category’s mean (i.e., from the intercept). The reference categories for each data set are reported in Table 5. The combination of these reference categories was predicted to yield the greatest anxiolytic effect of SSRIs and was therefore chosen. Note that in penalized regression, predictors are usually standardized. However, the effect of standardized dummies cannot be meaningfully interpreted. Therefore, only continuous predictors were standardized in this analysis. This may give dummy variables a slight advantage, leading them to become significant sooner than continuous ones.

**Table 5:** Reference categories for categorical moderators per anxiety test. EPM = elevated plus maze; MB = marble burying; USV = ultrasonic vocalization; SIH = stress-induced hyperthermia

	Reference category			
	EPM	MB	USV	SIH
Type of SSRI	Fluoxetine	Fluoxetine	Fluoxetine	Fluoxetine
Duration of treatment	Chronic	Chronic	Chronic	Chronic
Disease induction	Stress	Stress	Healthy	Healthy
Species	Rat	Mouse	Rat	Mouse
Sex	Male	Male	Both	Male
Use of pretest	No	No	No	No
Type of USV	-	-	Separation-induced	-
Type of SIH	-	-	-	Individual

Publication bias was assessed via visual inspection of funnel plots, and analysis of the Egger’s regression test and the likelihood ratio test (Satish and Joel 1988). Funnel plots were created by plotting the standardized mean difference (SMD) against the standard error. Asymmetry of the funnel plots was taken as a risk for publication bias and assessed by visual inspection. Egger’s regression test is a linear regression of the effect sizes on their standard errors, weighted by their inverse variance. A regression slope of zero is expected in the absence of publication bias (Lin and Chu 2018). We conducted likelihood ratio tests (LRT) to examine whether there was evidence that publications with a p-value < 0.05 were more likely to be published. Note that these methods for detecting publication bias are only valid for random effects models; they thus ignore the multilevel structure of the data.

## Results

### Data documentation

The Workflow for Open Reproducible Code in Science (WORCS) (Van Lissa et al. 2021) was used to make all analysis code and synthetic data available in a reproducible GitHub repository (10.17605/OSF.IO/3WD8C). A complete overview of the study characteristics of all included studies can be found in Supplementary file S2.

### Study selection

**Fig. 1:**
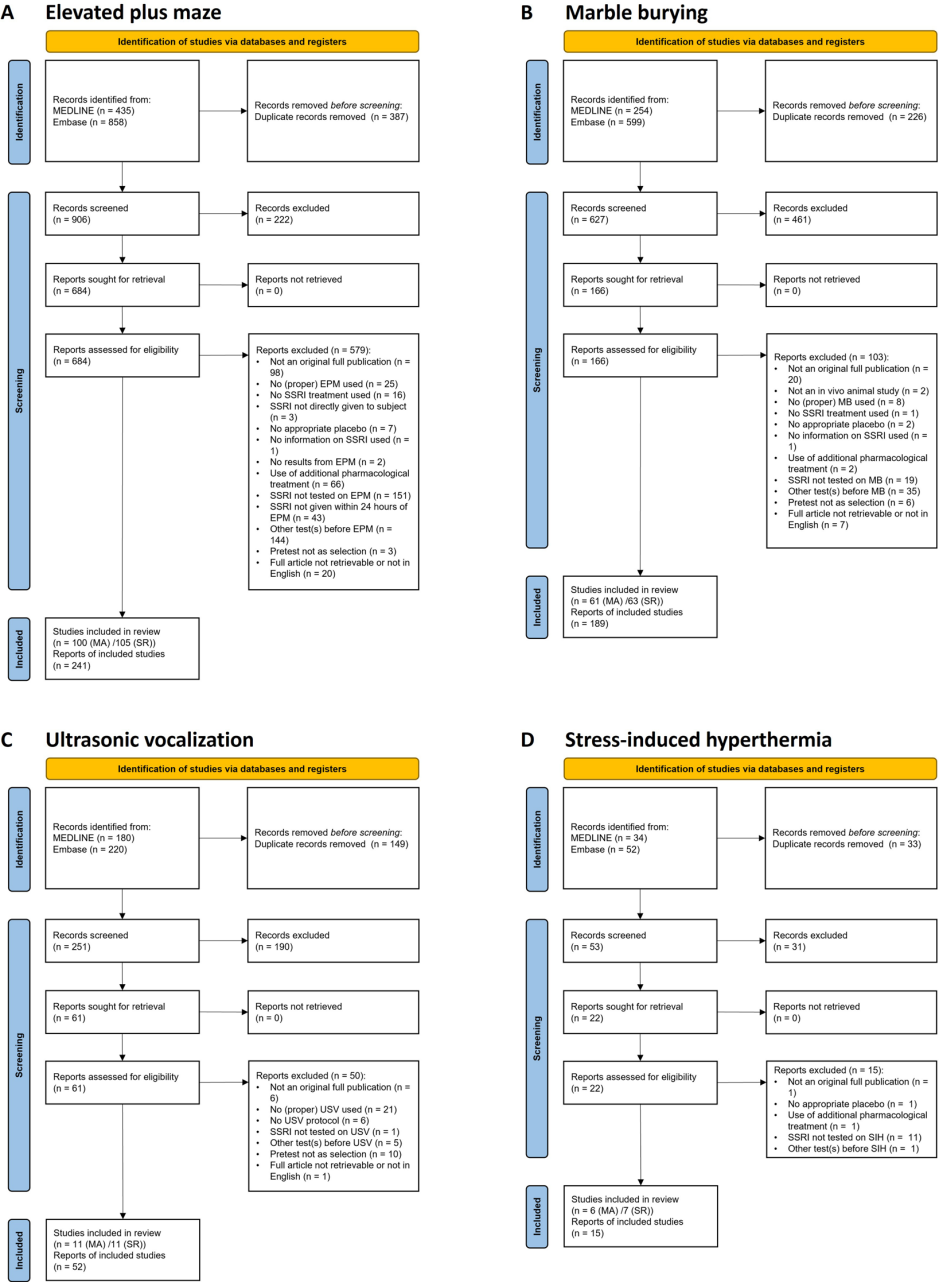
Flowcharts of the study selection process for the elevated plus maze (**A**), marble burying (**B**), ultrasonic vocalization (**C**), and stress-induced hyperthermia (**D**) tests. EPM = elevated plus maze, MB = marble burying, USV = ultrasonic vocalization, SIH = stress-induced hyperthermia, MA = meta-analysis, SR = systematic review

The Medline and Embase databases were systematically searched for relevant literature, which yielded 906 unique articles for the elevated plus maze, 627 unique articles for the marble burying, 251 unique articles for the ultrasonic vocalizations and 53 unique articles for the stress-induced hyperthermia. After full-text screening, the number of articles included in the systematic review/meta-analysis, respectively, was: 105/100 (elevated plus maze), 63/61 (marble burying), 11/11 (ultrasonic vocalizations) and 7/6 (stress-induced hyperthermia). The detailed selection procedures per anxiety test are visualized in flowcharts (Fig. 1).

### Summary of effects of SSRIs on unconditioned anxiety

**Fig. 2:**
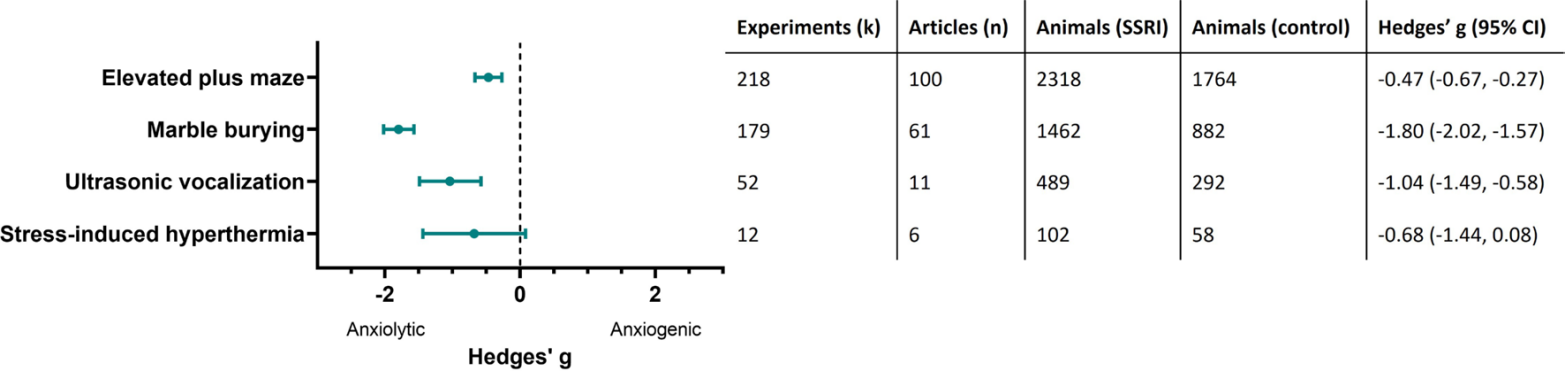
Pooled effect sizes (Hedges’ g and 95% CI) of SSRIs in the four anxiety tests. For each anxiety test the number of experiments (k), articles (n) animals in the intervention group (SSRI) and control group and the pooled effect sizes (Hedges’ g and 95% CI) are reported

**Fig. 3:**
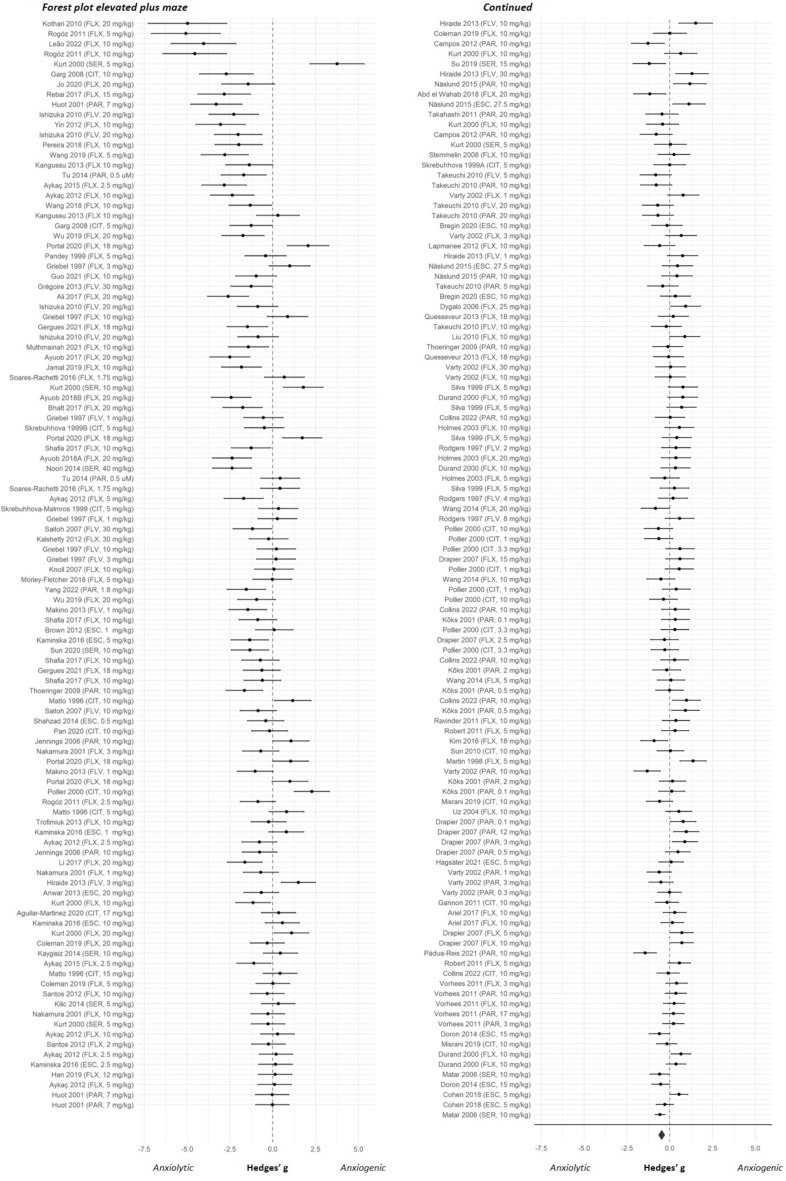
Forest plot of individual studies from the elevated plus maze dataset. Studies are ranked by their sampling variance, thus the most precise estimates are at the bottom of the plot. The overall effect was significant and favoured SSRI treatment (effect size (95% CI) 0.47 (0.67, 0.27); *p* < 0.001). CIT = citalopram, ESC = escitalopram, FLX = fluoxetine, FLV = fluvoxamine, PAR = paroxetine, SER = sertraline

**Fig. 4:**
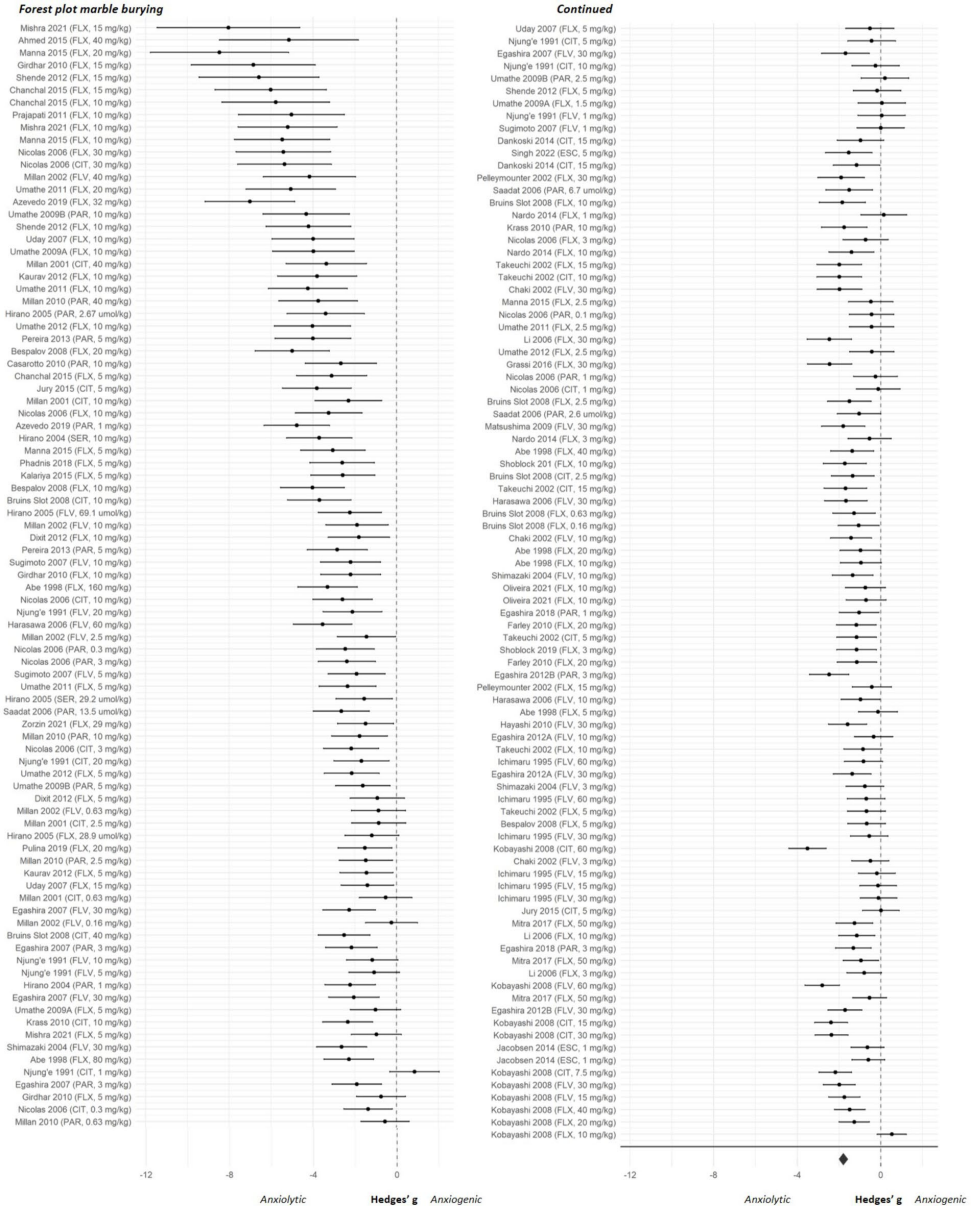
Forest plot of the individual studies from the marble burying dataset. Studies are ranked by their sampling variance, thus the most precise estimates are at the bottom of the plot. The overall effect was significant and favoured SSRI treatment (effect size (95% CI) -1.80 (-2.02, -1.57); *p* < 0.001). CIT = citalopram, ESC = escitalopram, FLX = fluoxetine, FLV = fluvoxamine, PAR = paroxetine, SER = sertraline

**Fig. 5:**
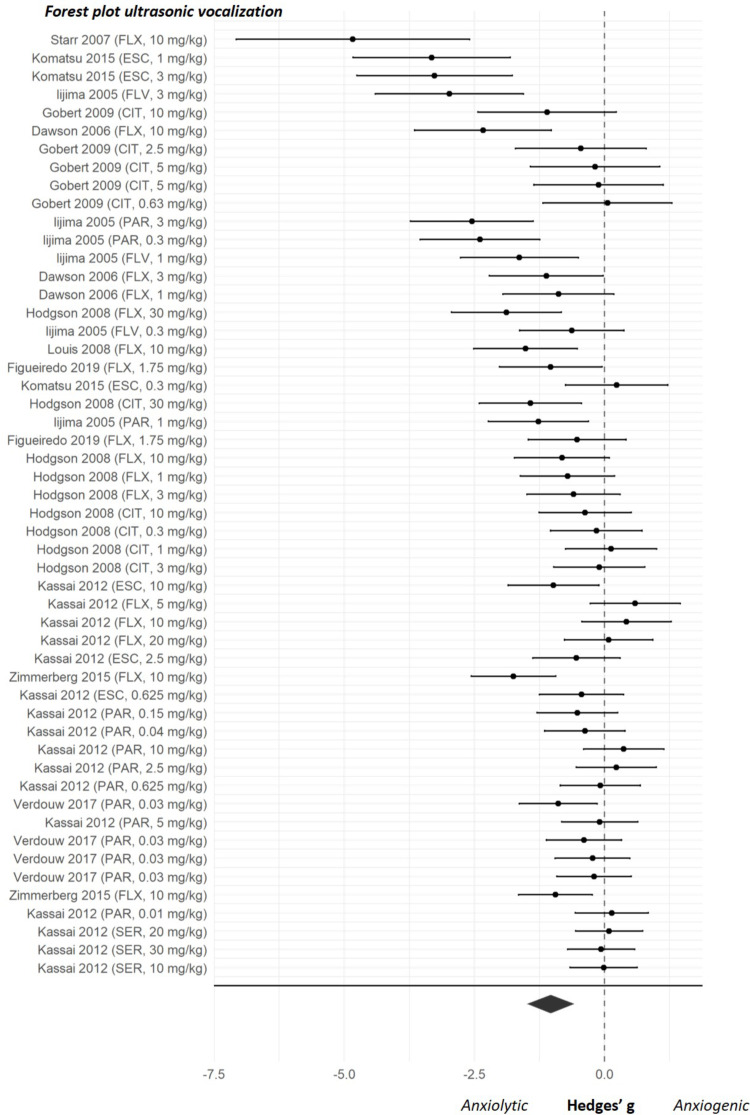
Forest plot of individual studies from the ultrasonic vocalization dataset. Studies are ranked by their sampling variance, thus the most precise estimates are at the bottom of the plot. The overall effect was significant and favoured SSRI treatment (effect size (95% CI) -1.04 (-1.49, -0.58); *p* < 0.001). CIT = citalopram, ESC = escitalopram, FLX = fluoxetine, FLV = fluvoxamine, PAR = paroxetine, SER = sertraline

**Fig. 6:**
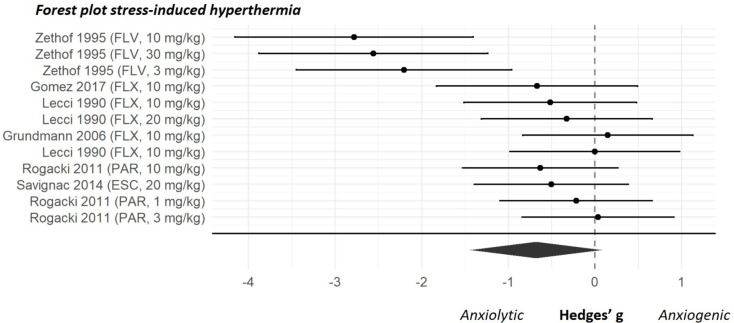
Forest plot of individual studies from the stress-induced hyperthermia dataset. Studies are ranked by their sampling variance, thus the most precise estimates are at the bottom of the plot. The overall effect was non-significant (effect size (95% CI) -0.68 (-1.44, 0.08); *p* = 0.08). Studies are ranked by their sampling variance, thus the most precise estimates are at the bottom of the plot. ESC = escitalopram, FLX = fluoxetine, FLV = fluvoxamine, PAR = paroxetine

The overall effect sizes of the four anxiety tests are shown in Fig. 2. Results of both the classical meta-regression and the BRMA can be found in Supplementary file S3. As shown in Fig. 2 the estimated pooled effect sizes of the four anxiety tests indicated that SSRIs reduce anxiety-like behaviour in the elevated plus maze, marble burying and ultrasonic vocalization tests but not in the stress-induced hyperthermia test. The forest plots showing the effect sizes of the individual experiments can be found in Figs. 3, 4, 5 and 6. Below, we describe the descriptive statistics and the meta-analysis for each anxiety test separately. We only reported moderator effects whose 95% credible interval excluded zero. This interval is the Bayesian counterpart of statistical significance since there is a 95% probability that it contains the population effect size.

### Elevated plus maze

#### Descriptive statistics

The effect of SSRIs on the elevated plus maze test was investigated in 241 unique experiments (105 articles). Fluoxetine was the most studied SSRI (*n* = 60), the other SSRIs were studied to a lesser extent (Table 6). Acute SSRI administration (*n* = 48) and subchronic SSRI administration (*n* = 59) dosing regimens were most commonly tested. The studied subjects were mostly rats (*n* = 59) and mice (*n* = 43). The majority of the articles used healthy, wild-type, naïve and non-stressed subjects (*n* = 71) and 41 articles used stress models (e.g., single prolonged stress). Male subjects were used most (*n* = 84) whereas only 10 articles used both sexes and 8 articles used females as study subjects. One of the 105 articles used a pretest for the selection of the study subjects. A summary of the descriptive statistics can be found in Tables 6 and 7.

**Table 6:** Descriptive statistics of all included articles per anxiety test. Numbers correspond to the number of articles that studied the specific moderators. EPM = elevated plus maze; MB = marble burying; USV = ultrasonic vocalization; SIH = stress-induced hyperthermia; NA = not applicable

		EPM	MB	USV	SIH
Type of SSRI	*Citalopram*	13	10	2	0
	*Escitalopram*	9	2	2	1
	*Fluoxetine*	60	37	7	3
	*Fluvoxamine*	8	15	1	1
	*Paroxetine*	16	13	3	2
	*Sertraline*	7	2	1	0
Duration of treatment	*Acute*	48	55	10	6
	*Subchronic*	59	3	2	1
	*Chronic*	7	11	0	1
Disease induction	*Healthy*	71	60	11	7
	*Stress*	41	4	0	0
	*Other*	13	3	1	0
Species	*Rat*	59	0	10	1
	*Mouse*	43	63	1	6
	*Gerbil*	1	0	0	0
	*Frog*	1	0	0	0
	*Hamster*	1	0	0	0
Sex	*Male*	84	54	2	7
	*Female*	8	3	1	0
	*Both*	10	4	5	0
	*NR*	4	2	2	0
Use of pretest	*Yes*	1	5	4	0
	*No*	104	58	7	7
Type of USV	*Separation-induced*	*NA*	*NA*	8	*NA*
	*Physical stress-induced*	*NA*	*NA*	3	*NA*
Type of SIH	*Individual*	*NA*	*NA*	*NA*	5
	*Group*	*NA*	*NA*	*NA*	2

**Table 7:** Studied dose ranges (mg/kg) of SSRIs in mg/kg per species in the included articles grouped per anxiety test. EPM = elevated plus maze; MB = marble burying; USV = ultrasonic vocalization; SIH = stress-induced hyperthermia

		EPM	MB	USV	SIH
Citalopram	*Rats*	1–17		0.3–30	
	*Mice*	5–10	0.3–60		
	*Hamster*	10			
Escitalopram	*Rats*	0.5–27.5		0.3–10	
	*Mice*	10–20	1–5		20
Fluoxetine	*Rats*	1–30		1–30	10
	*Mice*	1–30	0.16–160		10–20
	*Gerbils*	1–30			
	*Frogs*	5–20			
Fluvoxamine	*Rats*	1–30		0.3–3	
	*Mice*	1–20	0.16–60		3–30
Paroxetine	*Rats*	0.1–17		0.01–10	
	*Mice*	5–20	0.1–40	0.03	0.3–10
	*Gerbils*	0.3–10			
Sertraline	*Rats*	5–40			
	*Mice*	5–10	10		

#### Meta-analysis

Meta-analysis showed that SSRIs significantly reduced anxiety-like behaviour in the elevated plus maze test (effect size (95% CI) 0.47 (0.67, 0.27); *p* < 0.001)(Fig. 3). Significant within-study and between-study heterogeneity was observed as indicated by significant within and between-study variance, σ^2^_w_ 0.15 (0.05, 0.28) and σ^2^_b_ 0.71 (0.46, 1.09), respectively. The planned moderator analyses that we performed to identify sources of heterogeneity suggested that the effect of SSRIs on anxiety-like behaviour is dependent on the disease state of the animals. SSRIs had a significantly larger anxiolytic effect in stressed subjects than in healthy, wild-type, naïve and non-stressed subjects (Supplementary file S4).

### Marble burying

#### Descriptive statistics

The effects of SSRIs on marble burying were tested in 189 unique experiments (63 articles). Of these 63 articles, the majority used fluoxetine (*n* = 37). The other SSRIs had lower coverage (Table 6) and in most cases, SSRIs were administered acutely (*n* = 55). In all articles, mice were studied (*n* = 63) and the majority of these articles used healthy, wild-type, naïve and non-stressed mice (*n* = 60). In addition, most articles used male study subjects (*n* = 54) and only 3 articles used female subjects. All descriptive statistics of these experiments can be found in Tables 6 and 7.

#### Meta-analysis

Meta-analysis could be performed on 179 experiments. Results indicated that SSRIs significantly reduced anxiety-like behaviour in the marble burying test, with an effect size (95% CI) of -1.80 (-2.02, -1.57), *p* < 0.001 (Fig. 4). Significant between-study heterogeneity was observed as indicated by significant between-study variance, σ^2^_b_ 0.22 (0.00, 0.62), but no significant within-study heterogeneity was detected, σ^2^_w_ 1.05 (0.71, 1.52). Moderator analysis indicated that the human equivalent dose (HED) moderated the effect of SSRIs on anxiety-like behaviour. Significantly larger anxiolytic effects of SSRIs were observed with an increasing HED (SMD (95% CI) -0.35 (-0.49, -0.21))(Supplementary file S4).

### Ultrasonic vocalization

#### Descriptive statistics

The effects of SSRIs on ultrasonic vocalization were tested in 52 unique experiments (11 articles). Fluoxetine (*n* = 7), paroxetine (*n* = 3), citalopram (*n* = 2), escitalopram (*n* = 1) and fluvoxamine (*n* = 1) were studied and administered acutely (*n* = 10) in most articles. The studied subjects of 10 articles were rats and 1 article used mice as study subjects. Most of these articles used healthy, wild-type, naïve and non-stressed subjects (*n* = 11) and 1 article used study subjects that overexpressed corticotropin-releasing factor (CRF). Five articles used both sexes as study subjects, 2 articles used male study subjects and 1 article used female study subjects. Separation-induced ultrasonic vocalization was measured in 8 articles and physical stress-induced ultrasonic vocalization was assessed in 3 articles. A summary of the descriptive statistics can be found in Tables 6 and 7.

#### Meta-analysis

Meta-analysis showed that SSRIs significantly reduced anxiety-like behaviour in the ultrasonic vocalization test (effect size (95% CI) -1.04 (-1.49, -0.58); *p* < 0.001)(Fig. 5). Within-study heterogeneity was detected, indicated by significant within-study variance, σ^2^_w_ 0.12 (0.00, 0.36), but no between-study heterogeneity was observed, σ^2^_b_ 0.43 (0.12, 1.86). The moderator analysis did not identify any predictors for the SSRI effect in the ultrasonic vocalization test (Supplementary file S4).

### Stress-induced hyperthermia

#### Descriptive statistics

The effect of SSRIs on stress-induced hyperthermia was reported in 15 unique experiments (7 articles). Fluoxetine (*n* = 3), paroxetine (*n* = 2), escitalopram (*n* = 1) and fluvoxamine (*n* = 1) were studied. SSRIs were administered acutely (*n* = 6) in most of the articles. The studied subjects were mice (*n* = 6) and rats (*n* = 1). All 7 articles used healthy male study subjects, the stressor of the test was either rectal temperature measurement or open-field exposure. Five articles reported on an individual stress-induced hyperthermia test and 2 articles reported a group-housed stress-induced hyperthermia test. All descriptive statistics of these experiments are also shown in Tables 6 and 7.

#### Meta-analysis

Meta-analysis was performed on six articles and suggested that there was no significant effect of SSRIs on the anxiety response measured in the stress-induced hyperthermia test (effect size (95% CI) -0.68 (-1.44, 0.08); *p* = 0.08)(Fig. 6). Within-study heterogeneity was present, indicated by significant within-study variance, σ^2^_w_ 0.00 (0.00, 0.32), but no between-study heterogeneity was observed, σ^2^_b_ 0.73 (0.16, 4.07). Relevant predictors for the SSRI effect in the stress-induced hyperthermia test were not identified in the moderator analysis (Supplementary file S4).

### Locomotor activity

The effect of SSRIs on locomotor activity was reported in 114 experiments of the elevated plus maze dataset, 131 of the experiments of the marble burying dataset, and 11 of the experiments of the ultrasonic vocalization dataset. The majority of these experiments reported no significant effect of SSRIs on locomotor activity (elevated plus maze: *n* = 96 out of 114, marble burying: *n* = 117 out of 131, and ultrasonic vocalization: *n* = 9 out of 11). Many of the studies did not investigate the effect of SSRIs on locomotor activity at all (elevated plus maze: *n* = 75 out of 189, marble burying: *n* = 110 out of 231, and ultrasonic vocalization: *n* = 41 out of 52). All extracted locomotor activity data can be found in Supplementary file S5.

### Risk of bias assessment

**Fig. 7:**
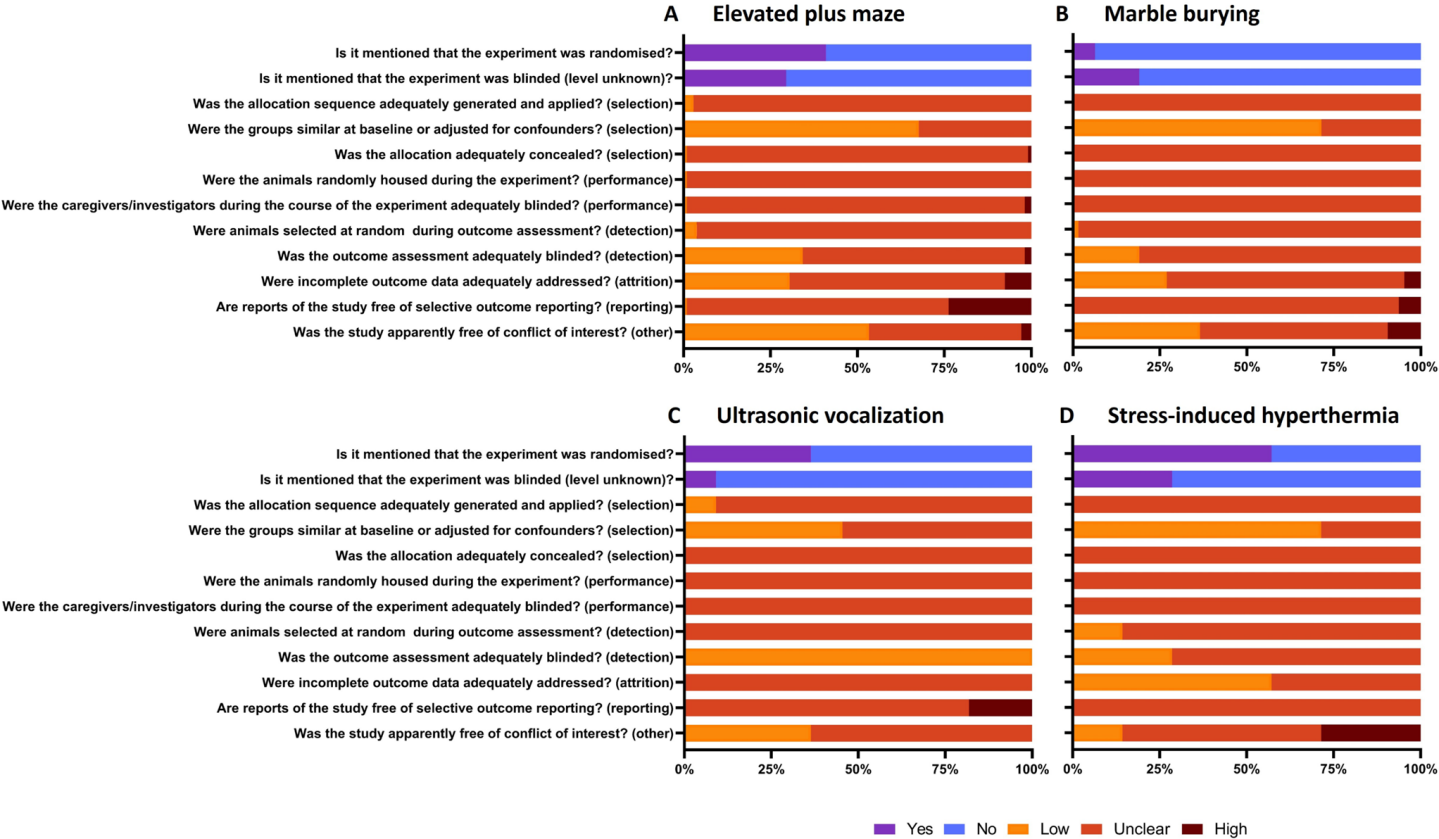
Risk of bias assessment of the elevated plus maze (**A**), the marble burying (**B**), the ultrasonic vocalization (**C**) and the stress-induced hyperthermia (**D**) tests

For all four anxiety tests the risk of bias categories were poorly reported in the respective articles, therefore the risk of bias was mostly scored as unclear. Below, we describe further details regarding the risk of bias assessment for each anxiety test separately (Fig. 7). The complete risk of bias assessment can be found in Supplementary file S2.

#### Elevated plus maze

In 59% of the articles, it was not mentioned whether the experiment was randomised and 70% of the articles did not mention whether the experiment was blinded. Furthermore, 24% of the articles showed selective outcome reporting. For example, they mentioned outcome measures in the method section which were not reported in the result section. On the other hand, groups were similar at baseline or adjusted for confounders in 68% of the articles and 53% of the articles stated that they were free of conflict of interest (Fig. 7A).

#### Marble burying

94% of the included articles did not mention whether the experiment was randomized and 81% of the articles did not mention whether the experiment was performed blinded. However, 71% of the articles used experimental groups that were similar at baseline or adjusted for confounders (Fig. 7B).

#### Ultrasonic vocalizations

64% of the articles did not mention randomization of the experiment and 91% of the articles did not mention whether the experiment was blinded. Also, 18% of the articles exhibited selective outcome reporting. On the other hand, all articles automated the outcome assessment by using video cameras and recording software, leading to a low risk of bias in terms of adequately blinding the outcome assessment (Fig. 7C).

#### Stress-induced hyperthermia

In 57% of the articles, it was mentioned that the experiment was randomized. However, 71% of the articles did not mention if the experiment was blinded. Groups were similar at baseline or were adjusted for confounders in 71% of the articles. In 57% of the articles, the incomplete outcome data was adequately addressed. A conflict of interest was reported by the authors in 29% of the articles (Fig. 7D).

### Publication bias

**Fig. 8:**
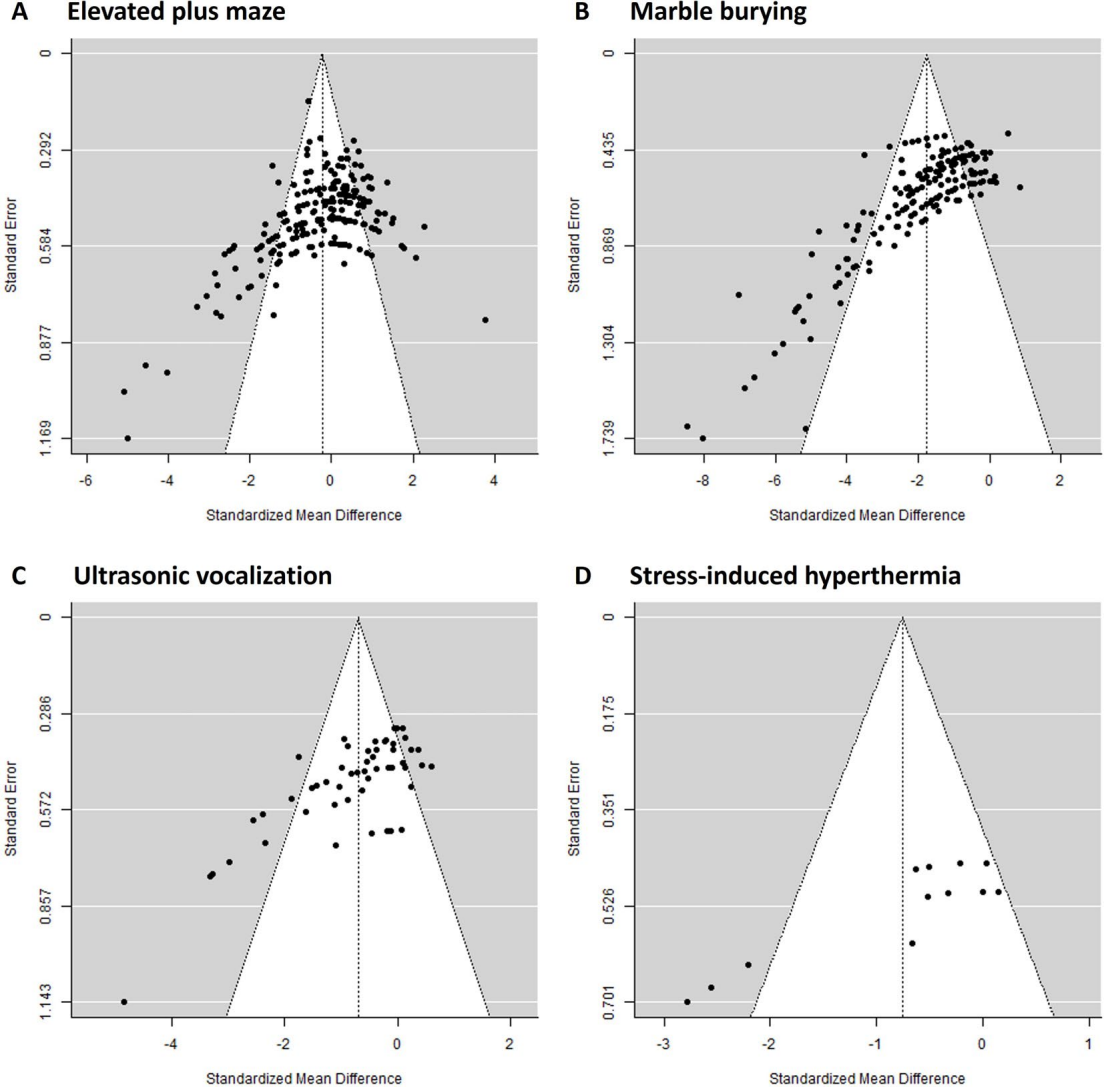
Publication bias visualized by funnel plots for the following unconditioned anxiety tests: (**A**) elevated plus maze, (**B**) marble burying, (**C**) ultrasonic vocalization, and (**D**) stress-induced hyperthermia. The vertical black dotted line represents the standardized mean difference (SMD) of the overall effect. Negative SMD values favour treatment. The most precise studies are at the top of the plot

Visual inspection of the funnel plots of the four unconditioned anxiety tests showed asymmetrical shapes and thus suggested the presence of publication bias in all four datasets (Fig. 8). The asymmetry of the funnel plots was confirmed by the Egger’s test. Results of this test indicated that publication bias was likely for all 4 data sets (elevated plus maze (Z = -8.54, *p* < 0.01), marble burying (Z = -14.95, *p* < 0.01), ultrasonic vocalization (Z = -7.11, *p* < 0.01), and stress-induced hyperthermia test (Z = -4.84, *p* < 0.01)). The likelihood ratio test indicated that for the marble burying (LRT(2) = 6.72, *p* < 0.01) and ultrasonic vocalization (LRT(2) = 6.80, *p* < 0.01) datasets papers with a p-value smaller than 0.05 were more likely to be published, whereas no evidence for such publication bias was found for the elevated plus maze (LRT(2) = 0.72, *p* = 0.40) and the stress-induced hyperthermia (LRT(2) = 1.15, *p* = 0.28) datasets.

## Discussion

With this review, we aimed to determine the effects of SSRIs on different aspects of unconditioned anxiety. For this, we analysed data from four animal tests for anxiety. Our meta-analysis indicated that SSRIs may reduce avoidance behaviour, burying behaviour and ultrasonic distress vocalizations. We found no evidence of an effect of SSRIs on stress-induced autonomic responses, which might be due to a lack of power. Moderator analysis showed that the effects of SSRIs on approach-avoidance behaviour were more pronounced in stressed animals than in control animals. In addition, the effect of SSRIs on burying behaviour was dose-dependent, with stronger beneficial effects observed with higher dosages. No moderating effect was observed for the type of SSRI, duration of treatment, species, sex, nor use of pre-test. Also, SSRIs do not seem to affect locomotor activity and thus the observed anxiolytic effect of SSRIs is not caused by non-specific effects.

### Critical evaluation of the analysed data

To properly evaluate and interpret the results of a meta-analysis, it is important to first assess the quality of the gathered data. The risk of bias and publication bias assessment revealed two major quality issues for all four analysed datasets. First, reporting of preventive measures to reduce the risk of bias was poor in most of the included articles. In many of the articles, it was not mentioned whether the experiments were randomized or blinded. Furthermore, details regarding the allocation sequence generation and concealment were not reported in the majority of the articles. Since for most articles it was unclear whether the preventive measures were not reported or not performed at all, we did not exclude any articles based on their risk of bias assessment. However, this choice could have considerable consequences for the interpretation of the generated results. As in human studies (Saltaji et al. 2018; Schulz et al. 1995), lack of randomization, blinding and allocation concealment may result in overestimation of the effect size in experimental animal studies as well. An analysis of multiple systematic reviews of animal studies found that the failure to randomize, perform a blind outcome assessment and conceal allocation, significantly increased the effect sizes in animal studies (Hirst et al. 2014). Therefore, the lack of transparency in the majority of the analysed articles regarding bias-reducing preventive measures is a reason to interpret the results with caution. Second, there was evidence of publication bias in all four datasets, as indicated by asymmetrical funnel plots and significant Egger’s regression tests. The funnel plot asymmetry could be caused by the overrepresentation of smaller, imprecise studies that favour treatment with SSRIs. This indication of publication bias suggests that the overall effects of SSRIs in our meta-analyses are most likely overestimated and thus emphasizes the importance of interpreting the results with caution. While systematic reviews are of great importance to create an objective overview of the published literature within a specific field of research, if that literature is biased, the results will be biased too. It would be of great value if researchers published their non-significant findings on SSRIs as well. Allowing us to better estimate the unbiased effect of these drugs on anxiety-related behaviour in the future. This emphasizes the advantage of the publication bias assessment within a systematic review compared to a narrative review and its importance, since it will give an estimation of how complete the analysed dataset is and thus indicates to what extent reliable conclusions can be drawn from the published literature. Thus, due to a lack of transparency regarding bias-preventing measures and the reasonable likelihood of publication bias within our systematic review, the results should be interpreted with considerable caution.

It is also important to be aware of the drawbacks of the unconditioned anxiety tests we studied. The elevated plus maze is the most commonly used animal anxiety test. As a result, multiple adaptions of the test exist, leading to inconsistent results and limited comparability due to high variability in methodologies between laboratories (Rodgers and Dalvi 1997). The marble burying test is often used to assess anxiety- and compulsive-like behaviour and to measure the anxiolytic and/or anti-compulsive effect of potential drugs in preclinical testing. However, the translational usefulness is questioned since the results of marble burying testing have been inconsistent and contradictory up to now (de Brouwer et al. 2019). To improve the translational value of the marble burying test it is, for example, suggested to make use of the two-zone configuration where marbles are only placed in one section of the arena. This two-zone paradigm allows for discrimination between anxiety-like and compulsive-like behaviour in this test since animals expressing compulsive-like behaviour should keep demonstrating repetitive burying behaviour even though there is a possibility to avoid this activity (de Brouwer et al. 2019). A drawback of the separation-induced ultrasonic vocalization test in infantile rodents is that the brains of these animals are not yet fully developed at this stage (Semple et al. 2013). Also, many factors can influence the number of USVs that are emitted by the infant rodents such as the temperature of the test environment, the state of satiation, or the presence of the mother or littermates in the recording area (Hofer and Shair 1978; Hofer and Shair 1992; Nelson and Alberts 2002). In addition, all three aforementioned anxiety tests involve locomotor activity. Readouts of elevated plus maze and marble burying tests are directly related to locomotor activity whereas ultrasonic vocalizations may be indirectly correlated with locomotor activity (Laplagne and Elias Costa 2016; Schwarting et al. 2007). When testing pharmacological agents in these tests, their sedative and motor effects should always be investigated to ensure that the effect measured is specific to anxiety and not due to side effects of the agent. In our dataset, 47% of the elevated plus maze studies, 69% of the marble burying studies, and 21% of the ultrasonic vocalization studies also tested the effect of SSRIs on motor activity (Supplementary file S5). A drawback of the stress-induced hyperthermia test is that the hyperthermic response observed in animals is the opposite of the decreased intestinal temperature observed in stressed healthy humans (Vinkers et al. 2013). Since stress leads to consistent body temperature changes in humans, body temperature is still considered a good physiological outcome measure of stress. As suggested by (Vinkers et al. 2013), the use of the stress-induced hyperthermia test could potentially provide early proof of concept of drug effectiveness and thus aid the development of anxiolytic drugs. Lastly, all four tests may detect anxiolytic drug effects upon acute drug administration. Acute beneficial treatment effects of SSRIs, however, do not resemble the clinical situation and could be considered a drawback of these tests (Lenze et al. 2005; Rickels et al. 2003). Testing drug effects in animals with a pre-existing anxiety condition could mimic the clinical situation and associated drug exposure effects more accurately. Our meta-analyses, however, did not detect moderator effects for ‘disease induction’ (except for the elevated plus maze) and ‘duration of treatment’. This is likely due to limited data availability, but may also suggest that these tests have limited predictive validity with regard to duration of treatment exposure.

### Effect of SSRIs on unconditioned anxiety

Bayesian regularized meta-regression suggested that based on the available data, SSRIs may reduce multiple unconditioned anxiety aspects in animals. Three of the investigated unconditioned anxiety tests show sensitivity to the anxiolytic effect of SSRIs, the implications of these results will be discussed here. Our study suggests that SSRIs may reduce avoidance behaviour in animals. Avoidance behaviour as observed in the elevated plus maze can be linked to the extensive avoidance of feared objects or situations which is typical for individuals with an anxiety-like disorder. In addition, the conflict between approaching and avoiding the open areas in the elevated plus maze is a familiar concept of opposite drives as seen in the psychodynamic theories of human anxiety. Our results also suggested that repetitive burying behaviour in animals might be reduced after SSRI treatment. This repetitive behaviour in rodents resembles the increased stereotype pattern behaviour seen in obsessive-compulsive disorder (OCD) patients. Furthermore, our meta-analysis suggests that SSRIs may reduce the ultrasonic distress vocalizations in animals. The 22-kHz and the 40-kHz ultrasonic vocalizations in rats and mice, respectively, are used to communicate a negative emotional state and are induced when the animal is subjected to aversive conditions (Brudzynski 2007; Litvin et al. 2007). These ultrasonic distress vocalizations thus represent the distress that is experienced when exposed to unavoidable aversive situations (Jelen et al. 2003), which is often experienced by patients with anxiety.

Together, this meta-analysis, and our previous meta-analysis that investigated the effect of SSRIs on conditioned fear (Heesbeen et al. 2023) suggest that SSRIs might reduce many anxiety aspects in animals. Clinical data demonstrates that SSRIs are effective in treating various anxiety-like disorders. This raises the question of whether the anxiolytic effect of SSRIs that is observed in animals and humans is selective for specific anxiety aspects or whether it results from a general inhibition of fear-related emotions. Emotional blunting is frequently observed in patients who are chronically treated with SSRIs. Patients who experience emotional blunting are not able to express their feelings, both verbally and non-verbally, particularly when discussing issues that would normally require some emotional engagement (Opbroek et al. 2002; Reinblatt and Riddle 2006; Sansone and Sansone 2010). Even though they experience less emotional pain than before the treatment, other emotions that are a part of everyday life are also experienced to a lesser extent (Price et al. 2009). Emotional blunting as a result of SSRI treatment has not been investigated in anxiety patients yet. Based on our results it could be interesting to investigate to what extent general inhibition of fear-related emotions is responsible for the anxiolytic effect of SSRIs in animals and humans.

A moderator analysis was performed to assess the impact of a predefined list of study characteristics on the overall heterogeneity found within these unconditioned anxiety tests. The effect of SSRIs seemed to be moderated by pre-existing anxiety conditions in the elevated plus maze and dose dependency in the marble burying test. Theoretically, these conditions are expected to moderate the beneficial effect of SSRIs in all tests but were only identified as moderators of the SSRI effect in these two tests. Also, the current meta-analysis found no evidence that one of the other predefined moderators (type of SSRI, duration of treatment, species, sex, and use of pretest) influenced the pooled effect in any of the four unconditioned anxiety tests. The absence of a moderating effect of the type of SSRI is not surprising considering that all six SSRIs are used to treat anxiety-like disorders such as generalized anxiety disorder, social anxiety disorder and obsessive-compulsive disorder albeit according to clinical guidelines or as off-label use (Sartori and Singewald 2019). However, the lack of a moderating effect of duration of treatment was not expected since SSRIs have a delayed onset of action in the clinical treatment of anxiety-like disorders (Lenze et al. 2005; Rickels et al. 2003). Interestingly, duration of treatment does moderate the effect of SSRIs on cued fear expression as shown in our previous systematic review (Heesbeen et al. 2023). This may suggest that the anxiolytic effect of SSRIs is achieved differently in conditioned and unconditioned anxiety. Given the limited availability of data on many of the moderators, the absence of moderating effects in the current analysis should be interpreted with caution. For example, the absence of a dose-dependent effect in three of the included tests can be explained by the experimental setups that were used in these three unconditioned anxiety tests. It may well be that the dose-dependent effect found for the marble burying test would have been observed in the other three tests if a higher number of dose-response experiments were performed. Thus, due to insufficient availability of data, the current moderator analysis does not allow us to gain more insight into which conditions or circumstances influence the effect of SSRIs on unconditioned anxiety.

### Future research

The main goal of our systematic review was to investigate the effect of SSRIs on multiple unconditioned anxiety aspects. Due to the low quality of the included studies, no reliable conclusions can be drawn concerning this. Therefore we want to emphasize that the main takeaway of this systematic review is that the quality of (animal) research should be improved by promoting and enforcing transparency regarding bias-reducing measures (e.g. blinding, randomization, and allocation sequencing). In addition, it should always be reported how many animals were used per experimental group and missing data points should always be adequately addressed. Also, the publication of all results regardless of significance should be encouraged to ensure a complete and unbiased body of evidence. The latter can be stimulated by implementing preregistration of (animal) studies which aids the design of proper research studies and promotes the reporting of all outcomes (Heinl et al. 2022). In addition, scientific journals could facilitate the publication of non-significant findings more. When these changes are implemented, we will be more confident that the results of future systematic reviews will reflect the population effect more accurately and thus more reliable conclusions can be drawn regarding the anxiolytic effect of SSRIs.

Additionally, increasing the quality of the execution and reporting of animal studies could aid the identification of new therapeutic targets for patients with anxiety-like disorders. If all studies were executed properly and reported regardless of significance there might have been tests of unconditioned anxiety that clearly did not show an anxiolytic effect of SSRIs. These tests could reflect the SSRI non-responder group and could be interesting to investigate potential anxiolytics for this patient group. Similarly, it could be reliably determined which tests of unconditioned anxiety are sensitive to the anxiolytic effect of SSRIs. These tests could be used to identify new pharmacological treatments that, for example, show an acute anxiolytic response in these SSRI-sensitive behavioural tests.

#### Knowledge gaps

First, the majority of the studied subjects in all four tests were male whereas females were only studied in a minority of the experiments. In humans however, women are more likely to develop anxiety disorder than men (Angst and Dobler-Mikola 1985; Bruce et al. 2005; Regier et al. 1990), and there is preliminary evidence that women respond differently to antidepressants such as SSRIs (Khan et al. 2005; Yang et al. 2011). The translational value of these animal studies would thus be improved by carrying out further experiments on female subjects. Second, fluoxetine was by far the most studied SSRI whereas the other SSRIs were only studied in a few articles. Therefore, the potential differences in efficacy between the six SSRIs could not be investigated properly in this systematic review. Third, only a few articles investigated the effect of SSRIs on stress-induced hyperthermia. Compared to the other unconditioned anxiety tests, the stress-induced hyperthermia test measures the autonomic response to stress instead of a behavioural response. No effect of SSRIs on stress-induced hyperthermia was observed. This suggests that SSRIs might not affect the autonomic stress response but only on behavioural responses to stress. However, very few studies investigated the effect of SSRIs on stress-induced hyperthermia and publication bias was present. Therefore more research is needed to investigate the effect of SSRIs on the autonomic stress response.

#### Limitations of the systematic review design

The results of this systematic review should be interpreted in light of the protocol’s limitations. First, during the full-text assessment, articles that performed other behavioural tests before the anxiety test of interest were excluded since the performance of animals can be dependent on the order of a test battery (Blokland et al. 2012). Order of reporting was used to determine the test order when a timeline was not available. This could have resulted in the inclusion of animals previously exposed to behavioural tests but also in the exclusion of animals that were naïve to behavioural testing. This uncertainty needs to be taken into account when interpreting the results. However, it is difficult to predict to what extent this occurred since we are not able to determine how many cases this concerns. Furthermore, the selection based on test order could have also contributed to the observed publication bias. Including studies in which the main focus was the effect of SSRIs on unconditioned anxiety will most likely increase the observed publication bias whereas studies that investigated the anxiolytic effect of SSRIs as a secondary focus will probably demonstrate less publication bias. The second limitation relates to the many different outcome measures of the elevated plus maze that were reported, we predefined a limited number of outcome parameters to reduce heterogeneity and prevent dependency within the dataset. This approach of outcome selection might have altered the overall effect and/or moderator effects. A third limitation pertains to the choice of study characteristics included in the moderator analysis. Some of the extracted study characteristics such as age of the animals and route of administration of the SSRI were not investigated. To reliably examine heterogeneity a relatively high number of experiments is needed per moderator. Since the investigated dataset was relatively small compared to the number of potentially relevant moderators, a selection of moderators was made that were deemed to be most relevant based on previous research (Riley et al. 2011). Thus, part of the remaining heterogeneity might have been explained by including additional study characteristics in the moderator analysis.

## Conclusion

On average, the published literature shows an anxiolytic effect of SSRIs on unconditioned anxiety aspects. These results combined with our previous systematic review on the effects of SSRIs on conditioned anxiety suggest a more general blunting of emotions by SSRIs. The contribution of emotional blunting to the anxiolytic effect of SSRIs should be investigated further. However, these results should be interpreted with substantial caution since the quality of evidence was low. Bias-reducing preventive measures were not reported in the majority of studies. We, therefore, recommend improving the quality of reporting and the conduct of research in animal studies to avoid bias from taking place. Furthermore, publication bias was present for all four unconditioned anxiety tests, suggesting that our analysed dataset was incomplete and thus preventing us from drawing reliable conclusions. To obtain an unbiased estimate of the potential effects of SSRIs, researchers ought to publish all relevant studies, regardless of significance. Preregistration and registered reports may help achieve this goal. Our systematic review demonstrates that even though a lot of research has been performed within this field using many laboratory animals, we are still very limited in drawing any conclusions on how SSRIs affect anxiety. Future research should thus focus on improving the design and the execution of animal studies, thereby enhancing their reliability. On the other hand, since the use of animal studies has thus far not provided conclusive results, the use of human studies could also be considered as a possible valuable alternative to the currently used animal studies.

### Electronic supplementary material

Below is the link to the electronic supplementary material.

Supplementary Material 1

Supplementary Material 2

Supplementary Material 3

Supplementary Material 4

Supplementary Material 5
