# Supplementary material for: The effect of SSRIs on unconditioned anxiety: a systematic review and meta-analysis of animal studies
Source: Psychopharmacology (Berl). 2024 Jul 9;241(9):1731–55. doi: 10.1007/s00213-024-06645-2 (PMC11339141; doi:10.1007/s00213-024-06645-2)
Supplement: Supplementary file 1 — Supplementary Material 1 [file 213_2024_6645_MOESM1_ESM.pdf]

The effect of SSRIs on unconditioned anxiety: a systematic review and meta-analysis of animal studies

Psychopharmacology

Elise J Heesbeen, Tatum van Kampen, P Monika Verdouw, Caspar van Lissa, Elisabeth Y Bijlsma, Lucianne Groenink

Corresponding author: Lucianne Groenink, l.groenink@uu.nl

Supplementary file S1. Search strategy of the systematic review demonstrating the three included components (SSRIs, four unconditioned anxiety tests and animals) for the two searched databases (PubMed and Embase).

SUPPLEMENTARY FILE S1

Search String Components

SSRI COMPONENT

Pubmed notation

(“Citalopram” [MeSH] OR “citalopram*” [tiab] OR “Cytalopram” [tiab] OR “Seropram” [tiab] OR “Escitalopram*” [tiab] OR “Celexa” [tiab] OR “Lu-10-171” [tiab] OR “Lu10171” [tiab] OR “Lexapro” [tiab] OR “Fluoxetine” [MeSH] OR “fluoxetin*” [tiab] OR “N-Methyl-gamma-(4-(trifluoromethyl)phenoxy)benzenepropanamine” [tiab] OR “Lilly-110140” [tiab] OR “Lilly 110140” [tiab] OR “Lilly110140” [tiab] OR “Sarafem” [tiab] OR “Prozac*” [tiab] OR “Fluvoxamine” [MeSH] OR “fluvoxamin*” [tiab] OR “Fluvoxadura” [tiab] OR “Luvox” [tiab] OR “Floxyfral” [tiab] OR “Fevarin” [tiab] OR “Dumirox” [tiab] OR “Faverin” [tiab] OR “Desiflu” [tiab] OR “DU-23000” [tiab] OR “DU 23000” [tiab] OR “DU23000” [tiab] OR “Paroxetine” [MeSH] OR “paroxetin*” [tiab] OR “Aropax*” [tiab] OR “BRL-29060” [tiab] OR “BRL 29060” [tiab] OR “BRL29060” [tiab] OR “FG-7051” [tiab] OR “FG 7051” [tiab] OR “FG7051” [tiab] OR “Seroxat” [tiab] OR “Paxil*” [tiab] OR “Sertraline” [MeSH] OR “sertralin*” [tiab] OR “Zoloft” [tiab] OR “Altruline” [tiab] OR “Lustral” [tiab] OR “Aremis” [tiab] OR “Besitran” [tiab] OR “Sealdin” [tiab] OR “Gladem” [tiab] OR “1 (3 dimethylaminopropyl) 1 (4 fluorophenyl) 1 3 dihydroisobenzofuran 5 carbonitrile” [tiab] OR “1 (3 dimethylaminopropyl) 1 (4 fluorophenyl) 5 phthalancarbonitrile” [tiab] OR “1 (3 dimethylaminopropyl) 1 3 dihydro 1 (4 fluorophenyl) isobenzofuran 5 carbonitrile” [tiab] OR “5 phthalancarbonitrile 1 (3 dimethylaminopropyl) 1 (4 fluorophenyl)” [tiab] OR “acelopam” [tiab] OR “adeprenal” [tiab] OR “apo-cital” [tiab] OR “aurex” [tiab] OR “ceform” [tiab] OR “cilopress” [tiab] OR “cinavol” [tiab] OR “ciprager” [tiab] OR “cipram” [tiab] OR “cipramil” [tiab] OR “cipraned” [tiab] OR “ciprotan” [tiab] OR “ciral” [tiab] OR “citabax” [tiab] OR “citacip” [tiab] OR “citagen” [tiab] OR “cital” [tiab] OR “citalec” [tiab] OR “citalich” [tiab] OR “citalon” [tiab] OR “citalonte” [tiab] OR “citalostad” [tiab] OR “citalox” [tiab] OR “citalvir” [tiab] OR “citapram” [tiab] OR “citaxin” [tiab] OR “citesint” [tiab] OR “citopam” [tiab] OR “citrol” [tiab] OR “citronil” [tiab] OR “dalsan” [tiab] OR “elopram” [tiab] OR “exenadil” [tiab] OR “frimaind” [tiab] OR “futuril” [tiab] OR “galopran” [tiab] OR “humorap” [tiab] OR “kaidor” [tiab] OR “kitapram” [tiab] OR “linisan” [tiab] OR “lopracil” [tiab] OR “lopraxer” [tiab] OR “loxopram” [tiab] OR “lu 10 171” [tiab] OR “lu 10171” [tiab] OR “lu10 171” [tiab] OR “lupram” [tiab] OR “malicon” [tiab] OR “nitalapram” [tiab] OR “oropram” [tiab] OR “percitale” [tiab] OR “pralotam” [tiab] OR “pram” [tiab] OR “pramital” [tiab] OR “prefucet” [tiab] OR “pricital” [tiab] OR “prisdal” [tiab] OR “psiconor” [tiab] OR “recital” [tiab] OR “renevil” [tiab] OR “return” [tiab] OR “ricap” [tiab] OR “ropramin” [tiab] OR “selon” [tiab] OR “sepram” [tiab] OR “seralgan” [tiab] OR “seregra” [tiab] OR “serital” [tiab] OR “seror” [tiab] OR “sintopram” [tiab] OR “sotovon” [tiab] OR “talam” [tiab] OR “talosin” [tiab] OR “unstress” [tiab] OR “varom” [tiab] OR “vesema” [tiab] OR “xadorek” [tiab] OR “zanipram” [tiab] OR “zd 211” [tiab] OR “zd211” [tiab] OR “zeclicid” [tiab] OR “zentius” [tiab] OR “zitolex” [tiab] OR “zyloram” [tiab] OR “cipralex” [tiab] OR “enlift ” [tiab] OR “entact” [tiab] OR “esciprex” [tiab] OR “esciprex distab” [tiab] OR “lu 26054 0” [tiab] OR “lu 260540” [tiab] OR “lu260540” [tiab] OR “premalex” [tiab] OR “prilect” [tiab] OR “seroplex” [tiab] OR “sipralexa” [tiab] OR “zecidec” [tiab] OR “zocital” [tiab] OR “3 (4 trifluoromethylphenoxy) n methyl 3 phenylpropylamine” [tiab] OR “3 n methyl 3 phenyl 3 (4 trifluoromethylphenoxy) propylamine” [tiab] OR “actan” [tiab] OR “adofen” [tiab] OR “afeksin” [tiab] OR “alzac 20” [tiab] OR “andep” [tiab] OR “andepin” [tiab] OR “ansilan” [tiab] OR “atd 20” [tiab] OR “auroken” [tiab] OR “auscap” [tiab] OR “bioxetin” [tiab] OR “captaton” [tiab] OR “compound 110140” [tiab] OR “daforin” [tiab] OR “dagrilan” [tiab] OR “depren” [tiab] OR “deprex*” [tiab] OR “deprizac” [tiab] OR “deproxin” [tiab] OR “diesan” [tiab] OR “digassim” [tiab] OR “elizac” [tiab] OR “exostrept” [tiab] OR “felicium” [tiab] OR “fldiss” [tiab] OR “flotinal” [tiab] OR “floxet” [tiab] OR “fluctin*” [tiab] OR “fludac” [tiab] OR “flufran” [tiab] OR “fluketin” [tiab] OR “flunil” [tiab] OR “flunirin” [tiab] OR “fluohexal” [tiab] OR “fluoksetin” [tiab] OR “fluoksetyna” [tiab] OR “fluox*” [tiab] OR “fluronin” [tiab] OR “flusac” [tiab] OR “flustad” [tiab] OR “flutin*” [tiab] OR “flux*” [tiab] OR “fluzac” [tiab] OR “fokeston” [tiab] OR “fontex” [tiab] OR “foxetin” [tiab] OR “foxtin” [tiab] OR “fropine” [tiab] OR “fuloren” [tiab] OR “gerozac” [tiab] OR “ladose” [tiab] OR “lanclic” [tiab] OR “lorien” [tiab] OR “lovan” [tiab] OR

“luramon” [tiab] OR “ly 110140” [tiab] OR “ly110140” [tiab] OR “magrilan” [tiab] OR “margrilan” [tiab] OR “meropan” [tiab] OR “modipran” [tiab] OR “mutan” [tiab] OR “n methyl 3 phenyl 3 (4 trifluoromethylphenoxy) propylamine” [tiab] OR “n methyl 3 phenyl 3 [(alpha trifluoro para tolyl) oxy] propylamine” [tiab] OR “nopres” [tiab] OR “nuzak” [tiab] OR “olena” [tiab] OR “oxactin” [tiab] OR “oxedep” [tiab] OR “phenylpropylamine n methyl 3 (4 trifluoromethylphenoxy)” [tiab] OR “plazeron” [tiab] OR “plinzene” [tiab] OR “portal ” [tiab] OR “pragmaten” [tiab] OR “prizma” [tiab] OR “proctin” [tiab] OR “prodep” [tiab] OR “prosac” [tiab] OR “prozamel” [tiab] OR “prozamin” [tiab] OR “prozep” [tiab] OR “prozit” [tiab] OR “psipax” [tiab] OR “qualisac” [tiab] OR “rapiflux” [tiab] OR “reconcile” [tiab] OR “reneuron” [tiab] OR “rowexetina” [tiab] OR “salipax” [tiab] OR “sanzur” [tiab] OR “sartuzin” [tiab] OR “selfemra” [tiab] OR “seromex” [tiab] OR “seronil” [tiab] OR “sinzac” [tiab] OR “sofelin” [tiab] OR “stephadilat-s” [tiab] OR “xeredien” [tiab] OR “zactin” [tiab] OR “zepax” [tiab] OR “zinovat” [tiab] OR “5 methoxy 1 [4 (trifluoromethyl) phenyl] 1 pentanone o (2 aminoethyl) oxime” [tiab] OR “5 methoxy 4 (trifluoromethyl) valerophenone o (2 aminoethyl) oxime” [tiab] OR “fluoxamine” [tiab] OR “fluroxamine” [tiab] OR “4 (4 fluorophenyl) 3 [(3 4 methylenedioxyphenoxy) methyl] piperidine” [tiab] OR “arketis” [tiab] OR “aroxat” [tiab] OR “brisdelle” [tiab] OR “brl 29060a” [tiab] OR “brl29060a” [tiab] OR “daparox” [tiab] OR “deroxat” [tiab] OR “dexorat” [tiab] OR “divarius” [tiab] OR “dropax” [tiab] OR “euplix” [tiab] OR “eutimil” [tiab] OR “frosinor” [tiab] OR “motivan” [tiab] OR “optipar” [tiab] OR “paluxetil” [tiab] OR “paluxon” [tiab] OR “paroc” [tiab] OR “parogen” [tiab] OR “parox*” [tiab] OR “paxan” [tiab] OR “paxtine” [tiab] OR “paxxet” [tiab] OR “pexeva” [tiab] OR “sereupin” [tiab] OR “setine” [tiab] OR “si 211103” [tiab] OR “si211103” [tiab] OR “solben ” [tiab] OR “syntopar” [tiab] OR “tagonis” [tiab] OR “1 methylamino 4 (3 4 dichlorophenyl) tetralin” [tiab] OR “4 (3 4 dichlorophenyl) 1 2 3 4 tetrahydro n methyl 1 naphthalenamine” [tiab] OR “4 (3 4 dichlorophenyl) 1 2 3 4 tetrahydro n methylnaphthalen 1 amine” [tiab] OR “adjuvin” [tiab] OR “atruline” [tiab] OR “cp 51974” [tiab] OR “cp 51974 01” [tiab] OR “cp 51974 1” [tiab] OR “cp 519741” [tiab] OR “cp51974” [tiab] OR “cp51974 01” [tiab] OR “cp51974 1” [tiab] OR “cp5197401” [tiab] OR “cp519741” [tiab] OR “dominum” [tiab] OR “doxime” [tiab] OR “fatral” [tiab] OR “fridep” [tiab] OR “lesefer” [tiab] OR “n methyl 4 (3 4 dichlorophenyl) 1 2 3 4 tetrahydro 1 naphthylamine” [tiab] OR “nudep” [tiab] OR “seltra” [tiab] OR “serad” [tiab] OR “sercerin” [tiab] OR “serlain” [tiab] OR “serlift” [tiab] OR “sertranex” [tiab] OR “sertranquil” [tiab] OR “sosser” [tiab] OR “tatig” [tiab] OR “tresleen” [tiab] OR “zolof*” [tiab] OR “zosert” [tiab])

EMBASE notation

(‘Citalopram’/exp OR ‘citalopram*’:ti,ab,kw OR ‘Cytalopram’:ti,ab,kw OR ‘Seropram’:ti,ab,kw OR ‘Escitalopram*’:ti,ab,kw OR ‘Celexa’:ti,ab,kw OR ‘Lu-10-171’:ti,ab,kw OR ‘Lu10171’:ti,ab,kw OR ‘Lexapro’:ti,ab,kw OR ‘Fluoxetine’/exp OR ‘fluoxetin*’:ti,ab,kw OR ‘N-Methyl-gamma-(4-(trifluoromethyl)phenoxy)benzenepropanamine’:ti,ab,kw OR ‘Lilly-110140’:ti,ab,kw OR ‘Lilly 110140’:ti,ab,kw OR ‘Lilly110140’:ti,ab,kw OR ‘Sarafem’:ti,ab,kw OR ‘Prozac*’:ti,ab,kw OR ‘Fluvoxamine’/exp OR ‘fluvoxamin*’:ti,ab,kw OR ‘Fluvoxadura’:ti,ab,kw OR ‘Luvox’:ti,ab,kw OR ‘Floxyfral’:ti,ab,kw OR ‘Fevarin’:ti,ab,kw OR ‘Dumirox’:ti,ab,kw OR ‘Faverin’:ti,ab,kw OR ‘Desiflu’:ti,ab,kw OR ‘DU-23000’:ti,ab,kw OR ‘DU 23000’:ti,ab,kw OR ‘DU23000’:ti,ab,kw OR ‘Paroxetine’/exp OR ‘paroxetin*’:ti,ab,kw OR ‘Aropax*’:ti,ab,kw OR ‘BRL-29060’:ti,ab,kw OR ‘BRL 29060’:ti,ab,kw OR ‘BRL29060’:ti,ab,kw OR ‘FG-7051’:ti,ab,kw OR ‘FG 7051’:ti,ab,kw OR ‘FG7051’:ti,ab,kw OR ‘Seroxat’:ti,ab,kw OR ‘Paxil*’:ti,ab,kw OR ‘Sertraline’/exp OR ‘sertralin*’:ti,ab,kw OR ‘Zoloft’:ti,ab,kw OR ‘Altruline’:ti,ab,kw OR ‘Lustral’:ti,ab,kw OR ‘Aremis’:ti,ab,kw OR ‘Besitran’:ti,ab,kw OR ‘Sealdin’:ti,ab,kw OR ‘Gladem’:ti,ab,kw OR ‘1 (3 dimethylaminopropyl) 1 (4 fluorophenyl) 1 3 dihydroisobenzofuran 5 carbonitrile’:ti,ab,kw OR ‘1 (3 dimethylaminopropyl) 1 (4 fluorophenyl) 5 phthalancarbonitrile’:ti,ab,kw OR ‘1 (3 dimethylaminopropyl) 1 3 dihydro 1 (4 fluorophenyl) isobenzofuran 5 carbonitrile’:ti,ab,kw OR ‘5 phthalancarbonitrile 1 (3 dimethylaminopropyl) 1 (4 fluorophenyl)’:ti,ab,kw OR ‘acelopam’:ti,ab,kw OR ‘adeprenal’:ti,ab,kw OR ‘apo-cital’:ti,ab,kw OR ‘aurex’:ti,ab,kw OR ‘ceform’:ti,ab,kw OR ‘cilopress’:ti,ab,kw OR ‘cinavol’:ti,ab,kw OR ‘ciprager’:ti,ab,kw OR ‘cipram’:ti,ab,kw OR ‘cipramil’:ti,ab,kw OR ‘cipraned’:ti,ab,kw OR ‘ciprotan’:ti,ab,kw OR ‘ciral’:ti,ab,kw OR ‘citabax’:ti,ab,kw OR ‘citacip’:ti,ab,kw OR ‘citagen’:ti,ab,kw OR ‘cital’:ti,ab,kw OR ‘citalec’:ti,ab,kw OR ‘citalich’:ti,ab,kw OR ‘citalon’:ti,ab,kw OR ‘citalonte’:ti,ab,kw OR ‘citalostad’:ti,ab,kw OR ‘citalox’:ti,ab,kw OR ‘citalvir’:ti,ab,kw OR ‘citapram’:ti,ab,kw OR

‘citaxin’:ti,ab,kw OR ‘citesint’:ti,ab,kw OR ‘citopam’:ti,ab,kw OR ‘citrol’:ti,ab,kw OR ‘citronil’:ti,ab,kw OR ‘dalsan’:ti,ab,kw OR ‘elopram’:ti,ab,kw OR ‘exenadil’:ti,ab,kw OR ‘frimaind’:ti,ab,kw OR ‘futuril’:ti,ab,kw OR ‘galopran’:ti,ab,kw OR ‘humorap’:ti,ab,kw OR ‘kaidor’:ti,ab,kw OR ‘kitapram’:ti,ab,kw OR ‘linisan’:ti,ab,kw OR ‘lopracil’:ti,ab,kw OR ‘lopraxer’:ti,ab,kw OR ‘loxopram’:ti,ab,kw OR ‘lu 10 171’:ti,ab,kw OR ‘lu 10171’:ti,ab,kw OR ‘lu10 171’:ti,ab,kw OR ‘lupram’:ti,ab,kw OR ‘malicon’:ti,ab,kw OR ‘nitalapram’:ti,ab,kw OR ‘oropram’:ti,ab,kw OR ‘percitale’:ti,ab,kw OR ‘pralotam’:ti,ab,kw OR ‘pram’:ti,ab,kw OR ‘pramital’:ti,ab,kw OR ‘prefucet’:ti,ab,kw OR ‘pricital’:ti,ab,kw OR ‘prisdal’:ti,ab,kw OR ‘psiconor’:ti,ab,kw OR ‘recital’:ti,ab,kw OR ‘renevil’:ti,ab,kw OR ‘return’:ti,ab,kw OR ‘ricap’:ti,ab,kw OR ‘ropramin’:ti,ab,kw OR ‘selon’:ti,ab,kw OR ‘sepram’:ti,ab,kw OR ‘seralgan’:ti,ab,kw OR ‘seregra’:ti,ab,kw OR ‘serital’:ti,ab,kw OR ‘seror’:ti,ab,kw OR ‘sintopram’:ti,ab,kw OR ‘sotovon’:ti,ab,kw OR ‘talam’:ti,ab,kw OR ‘talosin’:ti,ab,kw OR ‘unstress’:ti,ab,kw OR ‘varom’:ti,ab,kw OR ‘vesema’:ti,ab,kw OR ‘xadorek’:ti,ab,kw OR ‘zanipram’:ti,ab,kw OR ‘zd 211’:ti,ab,kw OR ‘zd211’:ti,ab,kw OR ‘zeclicid’:ti,ab,kw OR ‘zentius’:ti,ab,kw OR ‘zitolex’:ti,ab,kw OR ‘zyloram’:ti,ab,kw OR ‘cipralex’:ti,ab,kw OR ‘enlift ’:ti,ab,kw OR ‘entact’:ti,ab,kw OR ‘esciprex’:ti,ab,kw OR ‘esciprex distab’:ti,ab,kw OR ‘lu 26054 0’:ti,ab,kw OR ‘lu 260540’:ti,ab,kw OR ‘lu260540’:ti,ab,kw OR ‘premalex’:ti,ab,kw OR ‘prilect’:ti,ab,kw OR ‘seroplex’:ti,ab,kw OR ‘sipralexa’:ti,ab,kw OR ‘zecidec’:ti,ab,kw OR ‘zocital’:ti,ab,kw OR ‘3 (4 trifluoromethylphenoxy) n methyl 3 phenylpropylamine’:ti,ab,kw OR ‘3 n methyl 3 phenyl 3 (4 trifluoromethylphenoxy) propylamine’:ti,ab,kw OR ‘actan’:ti,ab,kw OR ‘adofen’:ti,ab,kw OR ‘afeksin’:ti,ab,kw OR ‘alzac 20’:ti,ab,kw OR ‘andep’:ti,ab,kw OR ‘andepin’:ti,ab,kw OR ‘ansilan’:ti,ab,kw OR ‘atd 20’:ti,ab,kw OR ‘auroken’:ti,ab,kw OR ‘auscap’:ti,ab,kw OR ‘bioxetin’:ti,ab,kw OR ‘captaton’:ti,ab,kw OR ‘compound 110140’:ti,ab,kw OR ‘daforin’:ti,ab,kw OR ‘dagrilan’:ti,ab,kw OR ‘depren’:ti,ab,kw OR ‘deprex*’:ti,ab,kw OR ‘deprizac’:ti,ab,kw OR ‘deproxin’:ti,ab,kw OR ‘diesan’:ti,ab,kw OR ‘digassim’:ti,ab,kw OR ‘elizac’:ti,ab,kw OR ‘exostrept’:ti,ab,kw OR ‘felicium’:ti,ab,kw OR ‘fldiss’:ti,ab,kw OR ‘flotinal’:ti,ab,kw OR ‘floxet’:ti,ab,kw OR ‘fluctin*’:ti,ab,kw OR ‘fludac’:ti,ab,kw OR ‘flufran’:ti,ab,kw OR ‘fluketin’:ti,ab,kw OR ‘flunil’:ti,ab,kw OR ‘flunirin’:ti,ab,kw OR ‘fluohexal’:ti,ab,kw OR ‘fluoksetin’:ti,ab,kw OR ‘fluoksetyna’:ti,ab,kw OR ‘fluox*’:ti,ab,kw OR ‘fluronin’:ti,ab,kw OR ‘flusac’:ti,ab,kw OR ‘flustad’:ti,ab,kw OR ‘flutin*’:ti,ab,kw OR ‘flux*’:ti,ab,kw OR ‘fluzac’:ti,ab,kw OR ‘fokeston’:ti,ab,kw OR ‘fontex’:ti,ab,kw OR ‘foxetin’:ti,ab,kw OR ‘foxtin’:ti,ab,kw OR ‘fropine’:ti,ab,kw OR ‘fuloren’:ti,ab,kw OR ‘gerozac’:ti,ab,kw OR ‘ladose’:ti,ab,kw OR ‘lanclic’:ti,ab,kw OR ‘lorien’:ti,ab,kw OR ‘lovan’:ti,ab,kw OR ‘luramon’:ti,ab,kw OR ‘ly 110140’:ti,ab,kw OR ‘ly110140’:ti,ab,kw OR ‘magrilan’:ti,ab,kw OR ‘margrilan’:ti,ab,kw OR ‘meropan’:ti,ab,kw OR ‘modipran’:ti,ab,kw OR ‘mutan’:ti,ab,kw OR ‘n methyl 3 phenyl 3 (4 trifluoromethylphenoxy) propylamine’:ti,ab,kw OR ‘n methyl 3 phenyl 3 [(alpha trifluoro para tolyl) oxy] propylamine’:ti,ab,kw OR ‘nopres’:ti,ab,kw OR ‘nuzak’:ti,ab,kw OR ‘olena’:ti,ab,kw OR ‘oxactin’:ti,ab,kw OR ‘oxedep’:ti,ab,kw OR ‘phenylpropylamine n methyl 3 (4 trifluoromethylphenoxy)’:ti,ab,kw OR ‘plazeron’:ti,ab,kw OR ‘plinzene’:ti,ab,kw OR ‘portal ’:ti,ab,kw OR ‘pragmaten’:ti,ab,kw OR ‘prizma’:ti,ab,kw OR ‘proctin’:ti,ab,kw OR ‘prodep’:ti,ab,kw OR ‘prosac’:ti,ab,kw OR ‘prozamel’:ti,ab,kw OR ‘prozamin’:ti,ab,kw OR ‘prozep’:ti,ab,kw OR ‘prozit’:ti,ab,kw OR ‘psipax’:ti,ab,kw OR ‘qualisac’:ti,ab,kw OR ‘rapiflux’:ti,ab,kw OR ‘reconcile’:ti,ab,kw OR ‘reneuron’:ti,ab,kw OR ‘rowexetina’:ti,ab,kw OR ‘salipax’:ti,ab,kw OR ‘sanzur’:ti,ab,kw OR ‘sartuzin’:ti,ab,kw OR ‘selfemra’:ti,ab,kw OR ‘seromex’:ti,ab,kw OR ‘seronil’:ti,ab,kw OR ‘sinzac’:ti,ab,kw OR ‘sofelin’:ti,ab,kw OR ‘stephadilat-s’:ti,ab,kw OR ‘xeredien’:ti,ab,kw OR ‘zactin’:ti,ab,kw OR ‘zepax’:ti,ab,kw OR ‘zinovat’:ti,ab,kw OR ‘5 methoxy 1 [4 (trifluoromethyl) phenyl] 1 pentanone o (2 aminoethyl) oxime’:ti,ab,kw OR ‘5 methoxy 4 (trifluoromethyl) valerophenone o (2 aminoethyl) oxime’:ti,ab,kw OR ‘fluoxamine’:ti,ab,kw OR ‘fluroxamine’:ti,ab,kw OR ‘4 (4 fluorophenyl) 3 [(3 4 methylenedioxyphenoxy) methyl] piperidine’:ti,ab,kw OR ‘arketis’:ti,ab,kw OR ‘aroxat’:ti,ab,kw OR ‘brisdelle’:ti,ab,kw OR ‘brl 29060a’:ti,ab,kw OR ‘brl29060a’:ti,ab,kw OR ‘daparox’:ti,ab,kw OR ‘deroxat’:ti,ab,kw OR ‘dexorat’:ti,ab,kw OR ‘divarius’:ti,ab,kw OR ‘dropax’:ti,ab,kw OR ‘euplix’:ti,ab,kw OR ‘eutimil’:ti,ab,kw OR ‘frosinor’:ti,ab,kw OR ‘motivan’:ti,ab,kw OR ‘optipar’:ti,ab,kw OR ‘paluxetil’:ti,ab,kw OR ‘paluxon’:ti,ab,kw OR ‘paroc’:ti,ab,kw OR ‘parogen’:ti,ab,kw OR ‘parox*’:ti,ab,kw OR ‘paxan’:ti,ab,kw OR ‘paxtine’:ti,ab,kw OR ‘paxxet’:ti,ab,kw OR ‘pexeva’:ti,ab,kw OR ‘sereupin’:ti,ab,kw OR ‘setine’:ti,ab,kw OR ‘si 211103’:ti,ab,kw OR ‘si211103’:ti,ab,kw OR ‘solben ’:ti,ab,kw OR ‘syntopar’:ti,ab,kw OR ‘tagonis’:ti,ab,kw OR ‘1 methylamino 4 (3 4 dichlorophenyl) tetralin’:ti,ab,kw OR ‘4 (3 4 dichlorophenyl) 1 2 3 4 tetrahydro n methyl 1 naphthalenamine’:ti,ab,kw OR ‘4 (3 4 dichlorophenyl) 1 2 3 4 tetrahydro n methylnaphthalen 1 amine’:ti,ab,kw OR ‘adjuvin’:ti,ab,kw OR ‘atruline’:ti,ab,kw OR ‘cp 51974’:ti,ab,kw OR ‘cp

51974 01':ti,ab,kw OR 'cp 51974 1':ti,ab,kw OR 'cp 519741':ti,ab,kw OR 'cp51974':ti,ab,kw OR 'cp51974 01':ti,ab,kw OR 'cp51974 1':ti,ab,kw OR 'cp5197401':ti,ab,kw OR 'cp519741':ti,ab,kw OR 'dominum':ti,ab,kw OR 'doxime':ti,ab,kw OR 'fatral':ti,ab,kw OR 'fridep':ti,ab,kw OR 'lesefer':ti,ab,kw OR 'n methyl 4 (3 4 dichlorophenyl) 1 2 3 4 tetrahydro 1 naphthylamine':ti,ab,kw OR 'nudep':ti,ab,kw OR 'seltra':ti,ab,kw OR 'serad':ti,ab,kw OR 'sercerin':ti,ab,kw OR 'serlain':ti,ab,kw OR 'serlift':ti,ab,kw OR 'sertranex':ti,ab,kw OR 'sertranquil':ti,ab,kw OR 'sosser':ti,ab,kw OR 'tatig':ti,ab,kw OR 'tresleen':ti,ab,kw OR 'zolof*':ti,ab,kw OR 'zosert':ti,ab,kw)

ELEVATED PLUS MAZE COMPONENT

Pubmed notation

("Elevated Plus Maze Test" [MeSH] OR "Elevated Plus Maze*" [tiab] OR "Maze, Elevated Plus" [tiab] OR "Plus Maze"[tiab] OR "x-maze" [tiab] OR "EPM" [tiab])

EMBASE notation

('Elevated Plus Maze Test'/exp OR 'Elevated Plus Maze*':ti,ab,kw OR 'Maze, Elevated Plus':ti,ab,kw OR 'Plus Maze':ti,ab,kw OR 'X-maze':ti,ab,kw OR 'EPM':ti,ab,kw)

MARBLE BURYING COMPONENT

Pubmed notation

("marble burying"[tiab] OR "burying test"[tiab] OR marble-burying[tiab] OR "burying behaviour"[tiab] OR "burying behavior"[tiab] OR "obsessive-compulsive disorder"[Mesh] OR OCD[tiab] OR "obsessive compulsive disorder"[tiab])

EMBASE notation

('marble burying test'/exp OR 'marble burying':ti,ab,kw OR 'burying test':ti,ab,kw OR 'marble-burying':ti,ab,kw OR 'marble burying behavior'/exp OR 'burying behaviour':ti,ab,kw OR 'burying behavior':ti,ab,kw OR 'obsessive-compulsive disorder':ti,ab,kw OR 'ocd':ti,ab,kw OR 'obsessive compulsive disorder':ti,ab,kw OR 'obsessive compulsive disorder'/exp)

ULTRASONIC VOCALISATION COMPONENT

Pubmed notation

("vocalization, animal" [MeSH Terms] OR vocalization* [tiab] OR vocalisation* [tiab])

EMBASE notation

('vocalization'/exp OR vocalization*:ti,ab,kw OR vocalisation*:ti,ab,kw)

STRESS-INDUCES HYPERTHERMIA COMPONENT

Pubmed notation

("Stress-induced hyperthermia*" [tiab] OR "SIH" [tiab] OR "Stress hyperthermia*" [tiab] OR "Emotional hyperthermia*" [tiab] OR "Psychogenic hyperthermia*" [tiab] OR "Psychological hyperthermia*" [tiab] OR "Emotional stress-induced hyperthermia*" [tiab] OR "Psychogenic stress-induced hyperthermia*" [tiab] OR "Psychological stress-induced hyperthermia*" [tiab] OR "PSH" [tiab] OR "Stress-induced thermal response*" [tiab] OR "Stress thermal response*" [tiab] OR "Emotional thermal response*" [tiab] OR "Psychogenic thermal response*" [tiab] OR "Psychological thermal response*" [tiab] OR "Stress-induced fever" [tiab] OR "Stress fever" [tiab] OR "Emotional fever" [tiab] OR "Psychogenic fever" [tiab] OR "Psychological fever" [tiab] OR "Stress-

induced changes in body temperature*” [tiab] OR “Stress changes in body temperature*” [tiab] OR “Emotional changes in body temperature*” [tiab] OR “Psychogenic changes in body temperature*” [tiab] OR “psychological changes in body temperature*” [tiab] OR “hyperthermia to emotional stress” [tiab])

Embase notation

(‘Stress-induced hyperthermia*’:ti,ab,kw OR ‘SIH’:ti,ab,kw OR ‘Stress hyperthermia*’:ti,ab,kw OR ‘Emotional hyperthermia*’:ti,ab,kw OR ‘Psychogenic hyperthermia*’:ti,ab,kw OR ‘Psychological hyperthermia*’:ti,ab,kw OR ‘Emotional stress-induced hyperthermia*’:ti,ab,kw OR ‘Psychogenic stress-induced hyperthermia*’:ti,ab,kw OR ‘Psychological stress-induced hyperthermia*’:ti,ab,kw OR ‘PSH’:ti,ab,kw OR ‘Stress-induced thermal response*’:ti,ab,kw OR ‘Stress thermal response*’:ti,ab,kw OR ‘Emotional thermal response*’:ti,ab,kw OR ‘Psychogenic thermal response*’:ti,ab,kw OR ‘Psychological thermal response*’:ti,ab,kw OR ‘Stress-induced fever’:ti,ab,kw OR ‘Stress fever’:ti,ab,kw OR ‘Emotional fever’:ti,ab,kw OR ‘Psychogenic fever’:ti,ab,kw OR ‘Psychological fever’:ti,ab,kw OR ‘Stress-induced changes in body temperature*’:ti,ab,kw OR ‘Stress changes in body temperature*’:ti,ab,kw OR ‘Emotional changes in body temperature*’:ti,ab,kw OR ‘Psychogenic changes in body temperature*’:ti,ab,kw OR ‘psychological changes in body temperature*’:ti,ab,kw OR ‘hyperthermia to emotional stress’:ti,ab,kw)

ANIMAL FILTER

Pubmed notation

(“animal experimentation”[MeSH Terms] OR “models, animal”[MeSH Terms] OR “invertebrates”[MeSH Terms] OR “Animals”[Mesh:noexp] OR “animal population groups”[MeSH Terms] OR “chordata”[MeSH Terms:noexp] OR “chordata, nonvertebrate”[MeSH Terms] OR “vertebrates”[MeSH Terms:noexp] OR “amphibians”[MeSH Terms] OR “birds”[MeSH Terms] OR “fishes”[MeSH Terms] OR “reptiles”[MeSH Terms] OR “mammals”[MeSH Terms:noexp] OR “primates”[MeSH Terms:noexp] OR “artiodactyla”[MeSH Terms] OR “carnivora”[MeSH Terms] OR “cetacea”[MeSH Terms] OR “chiroptera”[MeSH Terms] OR “elephants”[MeSH Terms] OR “hyraxes”[MeSH Terms] OR “insectivora”[MeSH Terms] OR “lagomorpha”[MeSH Terms] OR “marsupialia”[MeSH Terms] OR “monotremata”[MeSH Terms] OR “perissodactyla”[MeSH Terms] OR “rodentia”[MeSH Terms] OR “scandentia”[MeSH Terms] OR “sirenia”[MeSH Terms] OR “xenarthra”[MeSH Terms] OR “haplorhini”[MeSH Terms:noexp] OR “strepsirhini”[MeSH Terms] OR “platyrrhini”[MeSH Terms] OR “tarsii”[MeSH Terms] OR “catarrhini”[MeSH Terms:noexp] OR “cercopithecidae”[MeSH Terms] OR “hylobatidae”[MeSH Terms] OR “hominidae”[MeSH Terms:noexp] OR “gorilla gorilla”[MeSH Terms] OR “pan paniscus”[MeSH Terms] OR “pan troglodytes”[MeSH Terms] OR “pongo pygmaeus”[MeSH Terms]) OR ((animals[tiab] OR animal[tiab] OR mice[Tiab] OR mus[Tiab] OR mouse[Tiab] OR murine[Tiab] OR woodmouse[tiab] OR rats[Tiab] OR rat[Tiab] OR murinae[Tiab] OR muridae[Tiab] OR cottonrat[tiab] OR cottonrats[tiab] OR hamster[tiab] OR hamsters[tiab] OR cricetinae[tiab] OR rodentia[Tiab] OR rodent[Tiab] OR rodents[Tiab] OR pigs[Tiab] OR pig[Tiab] OR swine[tiab] OR swines[tiab] OR piglets[tiab] OR piglet[tiab] OR boar[tiab] OR boars[tiab] OR “sus scrofa”[tiab] OR ferrets[tiab] OR ferret[tiab] OR polecat[tiab] OR polecats[tiab] OR “mustela putorius”[tiab] OR “guinea pigs”[Tiab] OR “guinea pig”[Tiab] OR cavia[Tiab] OR callithrix[Tiab] OR marmoset[Tiab] OR marmosets[Tiab] OR cebuella[Tiab] OR hapale[Tiab] OR octodon[Tiab] OR chinchilla[Tiab] OR chinchillas[Tiab] OR gerbillinae[Tiab] OR gerbil[Tiab] OR gerbils[Tiab] OR jird[Tiab] OR jirds[Tiab] OR merione[Tiab] OR meriones[Tiab] OR rabbits[Tiab] OR rabbit[Tiab] OR hares[Tiab] OR hare[Tiab] OR diptera[Tiab] OR flies[Tiab] OR fly[Tiab] OR dipteral[Tiab] OR drosophila[Tiab] OR drosophilidae[Tiab] OR cats[Tiab] OR cat[Tiab] OR carus[Tiab] OR felis[Tiab] OR nematoda[Tiab] OR nematode[Tiab] OR nematodes[Tiab] OR sipunculida[Tiab] OR dogs[Tiab] OR dog[Tiab] OR canine[Tiab] OR canines[Tiab] OR canis[Tiab] OR sheep[Tiab] OR sheeps[Tiab] OR mouflon[Tiab] OR mouflons[Tiab] OR ovis[Tiab] OR goats[Tiab] OR goat[Tiab] OR capra[Tiab] OR capras[Tiab] OR rupicapra[Tiab] OR rupicapras[Tiab] OR chamois[Tiab] OR haplorhini[Tiab] OR monkey[Tiab] OR monkeys[Tiab] OR anthropoidea[Tiab] OR anthropoids[Tiab] OR saguinus[Tiab] OR tamarin[Tiab] OR tamarins[Tiab] OR leontopithecus[Tiab] OR hominidae[Tiab] OR ape[Tiab] OR apes[Tiab] OR “pan paniscus”[Tiab] OR bonobo[Tiab] OR bonobos[Tiab] OR “pan troglodytes”[Tiab] OR gibbon[Tiab] OR gibbons[Tiab] OR siamang[Tiab] OR siamangs[Tiab] OR nomascus[Tiab] OR symphalangus[Tiab] OR chimpanzee[Tiab] OR chimpanzees[Tiab] OR prosimian[Tiab] OR prosimians[Tiab] OR “bush baby”[Tiab] OR bush babies[Tiab] OR galagos[Tiab] OR galago[Tiab] OR pongidae[Tiab] OR gorilla[Tiab] OR gorillas[Tiab] OR “pongo pygmaeus”[Tiab] OR orangutan[Tiab] OR orangutans[Tiab] OR lemur[Tiab] OR lemurs[Tiab] OR lemuridae[Tiab] OR horse[Tiab] OR horses[Tiab] OR

equus[Tiab] OR cow[Tiab] OR calf[Tiab] OR bull[Tiab] OR chicken[Tiab] OR chickens[Tiab] OR gallus[Tiab] OR quail[Tiab] OR bird[Tiab] OR birds[Tiab] OR quails[Tiab] OR poultry[Tiab] OR poultries[Tiab] OR fowl[Tiab] OR fowls[Tiab] OR reptile[Tiab] OR reptilia[Tiab] OR reptiles[Tiab] OR snakes[Tiab] OR snake[Tiab] OR lizard[Tiab] OR lizards[Tiab] OR alligator[Tiab] OR alligators[Tiab] OR crocodile[Tiab] OR crocodiles[Tiab] OR turtle[Tiab] OR turtles[Tiab] OR amphibian[Tiab] OR amphibians[Tiab] OR amphibia[Tiab] OR frog[Tiab] OR frogs[Tiab] OR bombina[Tiab] OR salientia[Tiab] OR toad[Tiab] OR toads[Tiab] OR "epidalea calamita"[Tiab] OR salamander[Tiab] OR salamanders[Tiab] OR eel[Tiab] OR eels[Tiab] OR fish[Tiab] OR fishes[Tiab] OR pisces[Tiab] OR catfish[Tiab] OR catfishes[Tiab] OR siluriformes[Tiab] OR arius[Tiab] OR heteropneustes[Tiab] OR sheatfish[Tiab] OR perch[Tiab] OR perches[Tiab] OR percidae[Tiab] OR perca[Tiab] OR trout[Tiab] OR trouts[Tiab] OR char[Tiab] OR chars[Tiab] OR salvelinus[Tiab] OR minnow[Tiab] OR cyprinidae[Tiab] OR carps[Tiab] OR carp[Tiab] OR zebrafish[Tiab] OR zebrafishes[Tiab] OR goldfish[Tiab] OR goldfishes[Tiab] OR guppy[Tiab] OR guppies[Tiab] OR chub[Tiab] OR chubs[Tiab] OR tinca[Tiab] OR barbels[Tiab] OR barbus[Tiab] OR pimephales[Tiab] OR promelas[Tiab] OR "poecilia reticulata"[Tiab] OR mullet[Tiab] OR mullets[Tiab] OR eel[Tiab] OR eels[Tiab] OR seahorse[Tiab] OR seahorses[Tiab] OR mugil curema[Tiab] OR atlantic cod[Tiab] OR shark[Tiab] OR sharks[Tiab] OR catshark[Tiab] OR anguilla[Tiab] OR salmonid[Tiab] OR salmonids[Tiab] OR whitefish[Tiab] OR whitefishes[Tiab] OR salmon[Tiab] OR salmons[Tiab] OR sole[Tiab] OR solea[Tiab] OR lamprey[Tiab] OR lampreys[Tiab] OR pumpkinseed[Tiab] OR sunfish[Tiab] OR sunfishes[Tiab] OR tilapia[Tiab] OR tilapias[Tiab] OR turbot[Tiab] OR turbots[Tiab] OR flatfish[Tiab] OR flatfishes[Tiab] OR sciuridae[Tiab] OR squirrel[Tiab] OR squirrels[Tiab] OR chipmunk[Tiab] OR chipmunks[Tiab] OR suslik[Tiab] OR susliks[Tiab] OR vole[Tiab] OR voles[Tiab] OR lemming[Tiab] OR lemmings[Tiab] OR muskrat[Tiab] OR muskrats[Tiab] OR lemmus[Tiab] OR otter[Tiab] OR otters[Tiab] OR marten[Tiab] OR martens[Tiab] OR martes[Tiab] OR weasel[Tiab] OR badger[Tiab] OR badgers[Tiab] OR ermine[Tiab] OR mink[Tiab] OR minks[Tiab] OR sable[Tiab] OR sables[Tiab] OR gulo[Tiab] OR gulos[Tiab] OR wolverine[Tiab] OR wolverines[Tiab] OR mustela[Tiab] OR llama[Tiab] OR llamas[Tiab] OR alpaca[Tiab] OR alpacas[Tiab] OR camelid[Tiab] OR camelids[Tiab] OR guanaco[Tiab] OR guanacos[Tiab] OR chiroptera[Tiab] OR chiropteras[Tiab] OR bat[Tiab] OR bats[Tiab] OR fox[Tiab] OR foxes[Tiab] OR iguana[Tiab] OR iguanas[Tiab] OR xenopus laevis[Tiab] OR parakeet[Tiab] OR parakeets[Tiab] OR parrot[Tiab] OR parrots[Tiab] OR donkey[Tiab] OR donkeys[Tiab] OR mule[Tiab] OR mules[Tiab] OR zebra[Tiab] OR zebras[Tiab] OR shrew[Tiab] OR shrews[Tiab] OR bison[Tiab] OR bisons[Tiab] OR buffalo[Tiab] OR buffaloes[Tiab] OR deer[Tiab] OR deers[Tiab] OR bear[Tiab] OR bears[Tiab] OR panda[Tiab] OR pandas[Tiab] OR "wild hog"[Tiab] OR "wild boar"[Tiab] OR fitchew[Tiab] OR fitch[Tiab] OR beaver[Tiab] OR beavers[Tiab] OR jerboa[Tiab] OR jerboas[Tiab] OR capybara[Tiab] OR capybaras[Tiab]) NOT medline[sb])

Embase notation

'animal experiment'/exp OR 'animal model'/exp OR 'experimental animal'/exp OR 'transgenic animal'/exp OR 'male animal'/exp OR 'female animal'/exp OR 'juvenile animal'/exp OR 'animal'/de OR 'chordata'/de OR 'vertebrate'/de OR 'tetrapod'/de OR 'fish'/exp OR 'amniote'/de OR 'amphibia'/exp OR 'mammal'/de OR 'reptile'/exp OR 'sauropsid'/exp OR 'therian'/de OR 'monotremate'/exp OR 'placental mammals'/de OR 'marsupial'/exp OR 'euarchontoglires'/de OR 'afrotheria'/exp OR 'boreoeutheria'/exp OR 'laurasiatheria'/exp OR 'xenarthra'/exp OR 'primate'/de OR 'dermoptera'/exp OR 'glires'/exp OR 'scandentia'/exp OR 'haplorhini'/de OR 'prosimian'/exp OR 'simian'/de OR 'tarsiiform'/exp OR 'catarrhini'/de OR 'platyrrhini'/exp OR 'ape'/de OR 'cercopithecidae'/exp OR 'hominid'/de OR 'hylobatidae'/exp OR 'chimpanzee'/exp OR 'gorilla'/exp OR 'orang utan'/exp OR animal:ab,ti OR animals:ab,ti OR pisces:ab,ti OR fish:ab,ti OR fishes:ab,ti OR catfish:ab,ti OR catfishes:ab,ti OR sheatfish:ab,ti OR silurus:ab,ti OR arius:ab,ti OR heteropneustes:ab,ti OR clarias:ab,ti OR gariepinus:ab,ti OR 'fathead minnow':ab,ti OR 'fathead minnows':ab,ti OR pimephales:ab,ti OR promelas:ab,ti OR cichlidae:ab,ti OR trout:ab,ti OR trouts:ab,ti OR char:ab,ti OR chars:ab,ti OR salvelinus:ab,ti OR salmo:ab,ti OR oncorhynchus:ab,ti OR guppy:ab,ti OR guppies:ab,ti OR millionfish:ab,ti OR poecilia:ab,ti OR goldfish:ab,ti OR goldfishes:ab,ti OR carassius:ab,ti OR auratus:ab,ti OR mullet:ab,ti OR mullets:ab,ti OR mugil:ab,ti OR curema:ab,ti OR shark:ab,ti OR sharks:ab,ti OR cod:ab,ti OR cods:ab,ti OR gadus:ab,ti OR morhua:ab,ti OR carp:ab,ti OR carps:ab,ti OR cyprinus:ab,ti OR carpio:ab,ti OR killifish:ab,ti OR eel:ab,ti OR eels:ab,ti OR anguilla:ab,ti OR zander:ab,ti OR sander:ab,ti OR lucioperca:ab,ti OR stizostedion:ab,ti OR turbot:ab,ti OR turbots:ab,ti OR psetta:ab,ti OR flatfish:ab,ti OR flatfishes:ab,ti OR plaice:ab,ti OR pleuronectes:ab,ti OR platessa:ab,ti OR tilapia:ab,ti OR tilapias:ab,ti OR oreochromis:ab,ti OR sarotherodon:ab,ti OR 'common sole':ab,ti OR 'dover sole':ab,ti OR solea:ab,ti OR zebrafish:ab,ti OR zebrafishes:ab,ti OR danio:ab,ti OR rerio:ab,ti OR seabass:ab,ti OR dicentrarchus:ab,ti OR labrax:ab,ti OR morone:ab,ti OR lamprey:ab,ti OR lampreys:ab,ti OR

petromyzon:ab,ti OR pumpkinseed:ab,ti OR pumpkinseeds:ab,ti OR lepomis:ab,ti OR gibbosus:ab,ti OR herring:ab,ti OR clupea:ab,ti OR harengus:ab,ti OR amphibia:ab,ti OR amphibian:ab,ti OR amphibians:ab,ti OR anura:ab,ti OR salientia:ab,ti OR frog:ab,ti OR frogs:ab,ti OR rana:ab,ti OR toad:ab,ti OR toads:ab,ti OR bufo:ab,ti OR xenopus:ab,ti OR laevis:ab,ti OR bombina:ab,ti OR epidalea:ab,ti OR calamita:ab,ti OR salamander:ab,ti OR salamanders:ab,ti OR newt:ab,ti OR newts:ab,ti OR triturus:ab,ti OR reptilia:ab,ti OR reptile:ab,ti OR reptiles:ab,ti OR 'bearded dragon':ab,ti OR pogona:ab,ti OR vitticeps:ab,ti OR iguana:ab,ti OR iguanas:ab,ti OR lizard:ab,ti OR lizards:ab,ti OR 'anguis fragilis':ab,ti OR turtle:ab,ti OR turtles:ab,ti OR snakes:ab,ti OR snake:ab,ti OR aves:ab,ti OR bird:ab,ti OR birds:ab,ti OR quail:ab,ti OR quails:ab,ti OR coturnix:ab,ti OR bobwhite:ab,ti OR colinus:ab,ti OR virginianus:ab,ti OR poultry:ab,ti OR poultries:ab,ti OR fowl:ab,ti OR fowls:ab,ti OR chicken:ab,ti OR chickens:ab,ti OR gallus:ab,ti OR 'zebra finch':ab,ti OR taeniopygia:ab,ti OR guttata:ab,ti OR canary:ab,ti OR canaries:ab,ti OR serinus:ab,ti OR canaria:ab,ti OR parakeet:ab,ti OR parakeets:ab,ti OR grasskeet:ab,ti OR parrot:ab,ti OR parrots:ab,ti OR psittacine:ab,ti OR psittacines:ab,ti OR shelduck:ab,ti OR tadorna:ab,ti OR goose:ab,ti OR geese:ab,ti OR branta:ab,ti OR leucopsis:ab,ti OR woodlark:ab,ti OR lullula:ab,ti OR flycatcher:ab,ti OR ficedula:ab,ti OR hypoleuca:ab,ti OR dove:ab,ti OR doves:ab,ti OR geopelia:ab,ti OR cuneata:ab,ti OR duck:ab,ti OR ducks:ab,ti OR greylag:ab,ti OR graylag:ab,ti OR anser:ab,ti OR harrier:ab,ti OR 'circus pygargus':ab,ti OR 'red knot':ab,ti OR 'great knot':ab,ti OR calidris:ab,ti OR canutus:ab,ti OR godwit:ab,ti OR limosa:ab,ti OR lapponica:ab,ti OR meleagris:ab,ti OR gallopavo:ab,ti OR jackdaw:ab,ti OR corvus:ab,ti OR monedula:ab,ti OR ruff:ab,ti OR philomachus:ab,ti OR pugnax:ab,ti OR lapwing:ab,ti OR peewit:ab,ti OR plover:ab,ti OR vanellus:ab,ti OR swan:ab,ti OR cygnus:ab,ti OR columbianus:ab,ti OR bewickii:ab,ti OR gull:ab,ti OR chroicocephalus:ab,ti OR ridibundus:ab,ti OR albifrons:ab,ti OR 'great tit':ab,ti OR parus:ab,ti OR aythya:ab,ti OR fuligula:ab,ti OR streptopelia:ab,ti OR risoria:ab,ti OR spoonbill:ab,ti OR platalea:ab,ti OR leucorodia:ab,ti OR blackbird:ab,ti OR turdus:ab,ti OR merula:ab,ti OR 'blue tit':ab,ti OR cyanistes:ab,ti OR pigeon:ab,ti OR pigeons:ab,ti OR columba:ab,ti OR pintail:ab,ti OR anas:ab,ti OR starling:ab,ti OR sturnus:ab,ti OR owl:ab,ti OR 'athene noctua':ab,ti OR pochard:ab,ti OR ferina:ab,ti OR cockatiel:ab,ti OR nymphicus:ab,ti OR hollandicus:ab,ti OR skylark:ab,ti OR alauda:ab,ti OR tern:ab,ti OR sterna:ab,ti OR teal:ab,ti OR crecca:ab,ti OR oystercatcher:ab,ti OR haematopus:ab,ti OR ostralegus:ab,ti OR shrew:ab,ti OR shrews:ab,ti OR sorex:ab,ti OR araneus:ab,ti OR crocidura:ab,ti OR russula:ab,ti OR 'european mole':ab,ti OR talpa:ab,ti OR chiroptera:ab,ti OR bat:ab,ti OR bats:ab,ti OR eptesicus:ab,ti OR serotinus:ab,ti OR myotis:ab,ti OR dasycneme:ab,ti OR daubentonii:ab,ti OR pipistrelle:ab,ti OR pipistrellus:ab,ti OR cat:ab,ti OR cats:ab,ti OR felis:ab,ti OR catus:ab,ti OR feline:ab,ti OR dog:ab,ti OR dogs:ab,ti OR canis:ab,ti OR canine:ab,ti OR canines:ab,ti OR otter:ab,ti OR otters:ab,ti OR lutra:ab,ti OR badger:ab,ti OR badgers:ab,ti OR meles:ab,ti OR fitchew:ab,ti OR fitch:ab,ti OR foumart:ab,ti OR foulmart:ab,ti OR ferrets:ab,ti OR ferret:ab,ti OR polecat:ab,ti OR polecats:ab,ti OR mustela:ab,ti OR putorius:ab,ti OR weasel:ab,ti OR weasels:ab,ti OR fox:ab,ti OR foxes:ab,ti OR vulpes:ab,ti OR 'common seal':ab,ti OR phoca:ab,ti OR vitulina:ab,ti OR 'grey seal':ab,ti OR halichoerus:ab,ti OR horse:ab,ti OR horses:ab,ti OR equus:ab,ti OR equine:ab,ti OR equidae:ab,ti OR donkey:ab,ti OR donkeys:ab,ti OR mule:ab,ti OR mules:ab,ti OR pig:ab,ti OR pigs:ab,ti OR swine:ab,ti OR swines:ab,ti OR hog:ab,ti OR hogs:ab,ti OR boar:ab,ti OR boars:ab,ti OR porcine:ab,ti OR piglet:ab,ti OR piglets:ab,ti OR sus:ab,ti OR scrofa:ab,ti OR llama:ab,ti OR llamas:ab,ti OR lama:ab,ti OR glama:ab,ti OR deer:ab,ti OR deers:ab,ti OR cervus:ab,ti OR elaphus:ab,ti OR cow:ab,ti OR cows:ab,ti OR 'bos taurus':ab,ti OR 'bos indicus':ab,ti OR bovine:ab,ti OR bull:ab,ti OR bulls:ab,ti OR cattle:ab,ti OR bison:ab,ti OR bisons:ab,ti OR sheep:ab,ti OR sheeps:ab,ti OR 'ovis aries':ab,ti OR ovine:ab,ti OR lamb:ab,ti OR lambs:ab,ti OR mouflon:ab,ti OR mouflons:ab,ti OR goat:ab,ti OR goats:ab,ti OR capra:ab,ti OR caprine:ab,ti OR chamois:ab,ti OR rupicapra:ab,ti OR leporidae:ab,ti OR lagomorpha:ab,ti OR lagomorph:ab,ti OR rabbit:ab,ti OR rabbits:ab,ti OR oryctolagus:ab,ti OR cuniculus:ab,ti OR laprine:ab,ti OR hares:ab,ti OR lepus:ab,ti OR rodentia:ab,ti OR rodent:ab,ti OR rodents:ab,ti OR murinae:ab,ti OR mouse:ab,ti OR mice:ab,ti OR mus:ab,ti OR musculus:ab,ti OR murine:ab,ti OR 'wood mouse':ab,ti OR apodemus:ab,ti OR rat:ab,ti OR rats:ab,ti OR rattus:ab,ti OR norvegicus:ab,ti OR 'guinea pig':ab,ti OR 'guinea pigs':ab,ti OR cavia:ab,ti OR porcellus:ab,ti OR hamster:ab,ti OR hamsters:ab,ti OR mesocricetus:ab,ti OR cricetulus:ab,ti OR cricetus:ab,ti OR gerbil:ab,ti OR gerbils:ab,ti OR jird:ab,ti OR jirds:ab,ti OR meriones:ab,ti OR unguiculatus:ab,ti OR jerboa:ab,ti OR jerboas:ab,ti OR jaculus:ab,ti OR chinchilla:ab,ti OR chinchillas:ab,ti OR beaver:ab,ti OR beavers:ab,ti OR 'castor fiber':ab,ti OR 'castor canadensis':ab,ti OR sciuridae:ab,ti OR squirrel:ab,ti OR squirrels:ab,ti OR sciurus:ab,ti OR chipmunk:ab,ti

OR chipmunks:ab,ti OR marmot:ab,ti OR marmots:ab,ti OR marmota:ab,ti OR suslik:ab,ti OR susliks:ab,ti OR spermophilus:ab,ti OR cynomys:ab,ti OR cottonrat:ab,ti OR cottonrats:ab,ti OR sigmodon:ab,ti OR vole:ab,ti OR voles:ab,ti OR microtus:ab,ti OR myodes:ab,ti OR glareolus:ab,ti OR primate:ab,ti OR primates:ab,ti OR prosimian:ab,ti OR prosimians:ab,ti OR lemur:ab,ti OR lemurs:ab,ti OR lemuridae:ab,ti OR loris:ab,ti OR 'bush baby':ab,ti OR 'bush babies':ab,ti OR bushbaby:ab,ti OR bushbabies:ab,ti OR galago:ab,ti OR galagos:ab,ti OR anthropoidea:ab,ti OR anthropoids:ab,ti OR simian:ab,ti OR simians:ab,ti OR monkey:ab,ti OR monkeys:ab,ti OR marmoset:ab,ti OR marmosets:ab,ti OR callithrix:ab,ti OR cebuella:ab,ti OR tamarin:ab,ti OR tamarins:ab,ti OR saguinus:ab,ti OR leontopithecus:ab,ti OR 'squirrel monkey':ab,ti OR 'squirrel monkeys':ab,ti OR saimiri:ab,ti OR 'night monkey':ab,ti OR 'night monkeys':ab,ti OR 'owl monkey':ab,ti OR 'owl monkeys':ab,ti OR douroucoulis:ab,ti OR aotus:ab,ti OR 'spider monkey':ab,ti OR 'spider monkeys':ab,ti OR ateles:ab,ti OR baboon:ab,ti OR baboons:ab,ti OR papio:ab,ti OR 'rhesus monkey':ab,ti OR macaque:ab,ti OR macaca:ab,ti OR mulatta:ab,ti OR cynomolgus:ab,ti OR fascicularis:ab,ti OR 'green monkey':ab,ti OR 'green monkeys':ab,ti OR chlorocebus:ab,ti OR vervet:ab,ti OR vervets:ab,ti OR pygerythrus:ab,ti OR hominoidea:ab,ti OR ape:ab,ti OR apes:ab,ti OR hylobatidae:ab,ti OR gibbon:ab,ti OR gibbons:ab,ti OR siamang:ab,ti OR siamangs:ab,ti OR nomascus:ab,ti OR symphalangus:ab,ti OR hominidae:ab,ti OR orangutan:ab,ti OR orangutans:ab,ti OR pongo:ab,ti OR chimpanzee:ab,ti OR chimpanzees:ab,ti OR 'pan troglodytes':ab,ti OR bonobo:ab,ti OR bonobos:ab,ti OR 'pan paniscus':ab,ti OR gorilla:ab,ti OR gorillas:ab,ti OR troglodytes:ab,ti
