# Supplementary material for: The effect of SSRIs on unconditioned anxiety: a systematic review and meta-analysis of animal studies
Source: Psychopharmacology (Berl). 2024 Jul 9;241(9):1731–55. doi: 10.1007/s00213-024-06645-2 (PMC11339141; doi:10.1007/s00213-024-06645-2)
Supplement: Supplementary file 2 — Supplementary Material 2 [file 213_2024_6645_MOESM2_ESM.html]

Meta-analysis for the Effect of SSRIs on Anxiety


# Meta-analysis for the Effect of SSRIs on Anxiety

#### 13 June, 2023

# 1 Reproducibility

This Preregistration-As-Code uses the Workflow for Open Reproducible Code in Science (Van Lissa et al. 2020) to ensure reproducibility and transparency.
All code is available at https://github.com/cjvanlissa/meta\_anx\_ssri.git.

# 2 Preprocessing

First, we compute effect sizes using `metafor`.

```
dat[["id_experiment"]] <- as.integer(factor(dat$article))
dat[["id_es"]] <- 1:nrow(dat)
dat$paper <- dat$article
dat[c("study", "article")] <- NULL

# Remove cases for sensitivity analysis
if(sensitivity){
  dat <- dat[which(!dat$sensitivity == 1), ]
}

dat$Sample <- ordered(dat$test)
samples <- levels(dat$Sample)
dat[["test"]] <- NULL

dat <- escalc(measure = "SMD",
         n1i = dat$ncon,
         n2i = dat$nexp,
         m1i = dat$mcon,
         m2i = dat$mexp,
         sd1i = dat$sdcon,
         sd2i = dat$sdexp,
         data = dat)

dat <- dat[!is.na(dat$yi), ]
dat$yi <- dat$yi * dat$multiplier
dat[["multiplier"]] <- NULL
zscores <- scale(dat[["yi"]])
maxz <- max(abs(zscores))
#if(maxz > 3.3) stop("Potential outlier in Y-space")
dat <- dat[!abs(zscores) > 3.3, ]
saveRDS(dat, "dat.RData")
```

```
dat <- readRDS("dat.RData")
samples <- levels(dat$Sample)

mods <- c("ssri", "frequency", "disease", "species", "sex",
"hed", "pretested", "sih_test_type", "usv_test_type")
if(!all(mods %in% names(dat))) stop()
cat <- names(dat)[sapply(dat, inherits, "factor")]
cat <- cat[cat %in% mods]
```

# 3 Analysis

```
mlm <- lapply(samples, function(p){
  df <- dat[dat$Sample == p, ]
  model_pubbias <- rma(df$yi, vi = df$vi)
  pubbias_selmodel <- metafor::selmodel(model_pubbias, type ="stepfun", steps = c(.05))
  png(paste0("funnel_", p, ".png"))
  metafor::funnel(model_pubbias)
  dev.off()
  svg(paste0("funnel_", p, ".svg"))
  metafor::funnel(model_pubbias)
  dev.off()

  pubbias <- regtest(x = df$yi, vi = df$vi)
  #Conduct meta-analyses
  #model.mods <- rma.mv(yi, vi, random = list(~ 1 | id_experiment, ~ 1 | id_es), data=df) 
  model.full <- rma.mv(yi, vi, random = list(~ 1 | id_experiment, ~ 1 | id_es), data=df) 
  model.between_null <- rma.mv(yi, vi, random = list(~ 1 | id_experiment, ~ 1 | id_es), sigma2=c(NA,0), data = df) 
  model.within_null <- rma.mv(yi, vi, random = list(~ 1 | id_experiment, ~ 1 | id_es), sigma2=c(0,NA), data = df) 
model.both_null <- rma.mv(yi, vi, random = list(~ 1 | id_experiment, ~ 1 | id_es), sigma2=c(0,0), data = df) 

# Compute I2
W <- diag(1/df$vi)
X <- model.full$X
P <- W - W %*% X %*% solve(t(X) %*% W %*% X) %*% t(X) %*% W
I2 <- 100 * model.full$sigma2 / (sum(model.full$sigma2) + (model.full$k-model.full$p)/sum(diag(P)))
names(I2) <- c("I2_Between", "I2_Within")

#model.mods <- rma.mv(yi, vi, mods = as.formula(paste0("~ ", paste(moderators, collapse = " + "))), random = list(~ 1 | id_experiment, ~ 1 | id_es), data = df) 
#ggplot(, aes(x=d, colour=interventioncode))+geom_density()
#anova(model.full,rma.mv(yi, vi, mods = ~interventioncode, random = list(~ 1 | id_experiment, ~ 1 | id_es), data = df) ) 
aov_within <- anova(model.full,model.within_null) 
aov_between <- anova(model.full,model.between_null) 
aov_bothnull <- anova(model.full,model.both_null) 
aov_table <- data.frame(rbind(
c(aov_between$fit.stats.f[c(3:4, 1)], LRT = NA, p = NA),
c(aov_within$fit.stats.r[c(3:4, 1)], LRT = aov_within$LRT, p = aov_within$pval),
c(aov_between$fit.stats.r[c(3:4, 1)], LRT = aov_between$LRT, p = aov_between$pval),
c(aov_bothnull$fit.stats.r[c(3:4, 1)], LRT = aov_bothnull$LRT, p = aov_bothnull$pval)
))
rownames(aov_table) <- c("Three-level model", "Within-studies variance constrained", "Between-studies variance constrained", "Both variance components constrained")
write.csv(aov_table, paste0("threelevel_ma_", p, ".csv"))
confints <- confint(model.full)
#CHeck convergence of variance components:
#par(mfrow=c(2,1))
#plot.profile1 <- profile(model.full, sigma2=1)
#plot.profile2 <- profile(model.full, sigma2=2)

#Write forest plot to file
xname <- paste0("Hedges' g (", p, ")")
tmp <- df[order(df$vi, decreasing = TRUE), ]
tmp$paperlabel <- paste(tmp$paper, tmp$type_ssri_and_dose)
df_es <- data.frame(
    Study = ordered(tmp$paperlabel, levels = unique(tmp$paperlabel)),
    y = 1:nrow(tmp),
    es = tmp$yi,
    lb = tmp$yi - 1.96*sqrt(tmp$vi),
    ub = tmp$yi + 1.96*sqrt(tmp$vi),
    Sample = p)

dfoverall <- data.frame(x = c(model.full$ci.ub, model.full$b[1,1], model.full$ci.lb, model.full$b[1,1]),
                        y = c(max(df_es$y)+2, max(df_es$y)+2.5, max(df_es$y)+2, max(df_es$y)+1.5))
ylabsize = 10
# if(p == "Acq retr to ctx") ylabsize =1
pforest <- ggplot(data=df_es)+ 
  geom_point(aes(y=y, x=es))+ 
  geom_errorbarh(aes(y = y, xmin=lb, xmax=ub), height=.1)+
  
  # geom_point(data=data.frame(y = max(df_es$y)+2, es = model.full$b[1,1]), aes(y=y, x=es), size=4, shape = 15, fill = "black")+ 
  # geom_errorbarh(data = data.frame(y = max(df_es$y)+2, lb = model.full$ci.lb, ub = model.full$ci.ub), aes(y = y, xmin=lb, xmax=ub), height=.4, size = 2)+
  geom_polygon(data = dfoverall, aes(x = x, y = y))+
  scale_x_continuous(name=xname, limits = c(min(df_es$lb), min(c(10,max(df_es$ub)))))+
  scale_y_continuous(name = "", breaks=1:max(df_es$y), labels = df_es$Study, trans="reverse")+
  #adding a vertical line at the effect = 0 mark
  geom_vline(xintercept=0, color="black", linetype="dashed", alpha=.5)+
  geom_hline(yintercept=max(df_es$y)+1)+
  theme(axis.text.y = element_text(size=ylabsize))+
  theme_minimal()

ggsave(paste0("threelevel_ma_forest", p, ".png"), pforest, device = "png", height = (nrow(df)+2)*.2)
res = data.frame(Variance = c("Overall ES", "tau2 between", "tau2 within"),
                 rbind(c(estimate = model.full$b[1,1], ci.lb = model.full$ci.lb, ci.ub = model.full$ci.ub, model.full$pval),
                       c(confints[[1]]$random[1,], aov_table$p[3]),
                       c(confints[[2]]$random[1,], aov_table$p[2])))
res[nrow(res)+c(1:2), ] <- NA
res[[1]][nrow(res)-c(0,1)] <- names(I2)
res[[2]][nrow(res)-c(0,1)] <- I2
  list(mod = model.full,
    aov_table = aov_table,
       res = res,
    pubbias = pubbias,
    pubbias_sel = pubbias_selmodel)     
})
names(mlm) <- samples
saveRDS(mlm, "mlm.RData")

tb <- lapply(1:length(samples), function(i){
  tb <- mlm[[i]]$res
  tb$Sample <- samples[i]
  tb
})
tb <- do.call(rbind, tb)
tb$CI <- tidySEM::conf_int(lb = tb$ci.lb, ub = tb$ci.ub)
tb[c("ci.lb", "ci.ub")] <- NULL
names(tb) <- c("Parameter", "Estimate", "p", "Sample", "CI")
write.csv(tb, "tab_threelevel.csv", row.names = FALSE)
```

## 3.1 Descriptive statistics

The effect size estimates ranged from -8.46 to 3.76 (M=−1.05,SD=1.60M=−1.05,SD=1.60 ).

Several studies reported multiple effect sizes (1 - 16, with most reporting 1 effect size).

## 3.2 Publication bias

We examined publication bias using funnel plots, Egger’s test, and selection models, see Iyengar, S., & Greenhouse, J. B. (1988). Selection models and the file drawer problem. Statistical Science, 3(1), 109–117. ⁠https://doi.org/10.1214/ss/1177013012⁠

Note that these methods for detecting publication bias are only valid for random effects models;
they thus ignore the multilevel structure of the data.

We conducted likelihood ratio tests to examine whether there was evidence that publications with a p-value < .05 were more likely to be published.

```
out = NULL
for (i in samples) {
  out = c(out, knit_expand('selbias_template.Rmd'))
}
```

### 3.2.1 Pub bias EPM

According to the selection model, there was no evidence of publication bias, LRT(2) = 0.72, p = 0.40.

Egger’s test for funnel plot asymmetry showed significant evidence of publication bias, Z = -8.54, p < 0.01.

```
knitr::include_graphics("funnel_EPM.png")
```

### 3.2.2 Pub bias MB

According to the selection model, there was significant evidence of publication bias, LRT(2) = 6.72, p < 0.01.

Egger’s test for funnel plot asymmetry showed significant evidence of publication bias, Z = -14.95, p < 0.01.

```
knitr::include_graphics("funnel_MB.png")
```

### 3.2.3 Pub bias SIH

According to the selection model, there was no evidence of publication bias, LRT(2) = 1.15, p = 0.28.

Egger’s test for funnel plot asymmetry showed significant evidence of publication bias, Z = -4.84, p < 0.01.

```
knitr::include_graphics("funnel_SIH.png")
```

### 3.2.4 Pub bias USV

According to the selection model, there was significant evidence of publication bias, LRT(2) = 6.80, p < 0.01.

Egger’s test for funnel plot asymmetry showed significant evidence of publication bias, Z = -7.11, p < 0.01.

```
knitr::include_graphics("funnel_USV.png")
```

## 3.3 Threelevel Multilevel RMA

Meta-analysis was conducted in R (R Core Team 2021) using the R-packages `metafor` (Viechtbauer et al. 2010), and `pema` (Van Erp S. 2021).
To estimate overall effects, we used three-level meta-analysis to account for dependent effect sizes within studies (Van den Noortgate et al. 2015).
Let yjkyjk  denote the jj  observed effect sizes yy , originating from kk  studies.
The multi-level model is then given by the following equations:

yjkβjkθk=βjk+ϵjk=θk+wjk=δ+bkwhere ϵjkwhere wjkwhere bk∼N(0,σ2ϵjk)∼N(0,σ2w)∼N(0,σ2b)⎫⎭⎬⎪⎪yjk=βjk+ϵjkwhere ϵjk∼N(0,σϵjk2)βjk=θk+wjkwhere wjk∼N(0,σw2)θk=δ+bkwhere bk∼N(0,σb2)}

The first equation indicates that observed effect sizes are equal to the underlying population effect size, plus sampling error ϵjkϵjk . The second equation indicates that population effect sizes within studies are a function of a study-specific true effect size, plus within-study residuals wjkwjk . The third equation indicates that the distribution of study-specific true effect sizes are distributed around an overall mean effect, with between-study residuals bkbk .

Separate meta-analyses were conducted for each of the samples.
The overall pooled effect sizes were:

```
tb <- read.csv("tab_threelevel.csv", stringsAsFactors = FALSE)
datatable(tb, rownames= FALSE, options = list(
              "pageLength" = nrow(tb))) |>
  formatRound(columns=c('Estimate', 'p'), digits=2)
```

Show 10202550100 entries

Search:

| Parameter | Estimate | p | Sample | CI |
| --- | --- | --- | --- | --- |
| Overall ES | -0.47 | 0.00 | EPM | [-0.67, -0.27] |
| tau2 between | 0.71 | 0.00 | EPM | [0.46, 1.09] |
| tau2 within | 0.15 | 0.00 | EPM | [0.05, 0.28] |
| I2\_Within | 13.74 |  | EPM | [NA, NA] |
| I2\_Between | 67.21 |  | EPM | [NA, NA] |
| Overall ES | -1.80 | 0.00 | MB | [-2.02, -1.57] |
| tau2 between | 0.22 | 0.00 | MB | [0.00, 0.62] |
| tau2 within | 1.05 | 0.06 | MB | [0.71, 1.52] |
| I2\_Within | 65.41 |  | MB | [NA, NA] |
| I2\_Between | 13.93 |  | MB | [NA, NA] |
| Overall ES | -0.68 | 0.08 | SIH | [-1.44, 0.08] |
| tau2 between | 0.73 | 1.00 | SIH | [0.16, 4.07] |
| tau2 within | 0.00 | 0.00 | SIH | [0.00, 0.32] |
| I2\_Within | 0.00 |  | SIH | [NA, NA] |
| I2\_Between | 73.13 |  | SIH | [NA, NA] |
| Overall ES | -1.04 | 0.00 | USV | [-1.49, -0.58] |
| tau2 between | 0.43 | 0.07 | USV | [0.12, 1.86] |
| tau2 within | 0.12 | 0.00 | USV | [0.00, 0.36] |
| I2\_Within | 15.93 |  | USV | [NA, NA] |
| I2\_Between | 56.63 |  | USV | [NA, NA] |

Showing 1 to 20 of 20 entries

Previous1Next

The overall effect size estimate differed significantly from zero for EPM, MB, USV.

The within-studies variance component σ2wσw2  (between effect sizes) was significant for .

The between-studies variance σ2bσb2  was significant for .

## 3.4 Forest plots

The forest plots for the aforementioned three-level meta-analyses are presented below.
Within each plot, studies are ranked by their sampling variance vivi ;
thus, the most precise estimates are at the bottom, near the overall effect.

```
knitr::include_graphics(paste0("threelevel_ma_forest", samples[1], ".png"))
```

Figure 3.1: Forest plot for EPM

```
knitr::include_graphics(paste0("threelevel_ma_forest", samples[2], ".png"))
```

Figure 3.2: Forest plot for MB

```
knitr::include_graphics(paste0("threelevel_ma_forest", samples[3], ".png"))
```

Figure 3.3: Forest plot for SIH

```
knitr::include_graphics(paste0("threelevel_ma_forest", samples[4], ".png"))
```

Figure 3.4: Forest plot for USV

## 3.5 Moderator analyses

The effect of multiple moderators was investigated using meta-regression.
For categorical variables, dummies were encoded.
As the number of moderators is high relative to the number of studies,
there is a risk of overfitting and model non-identification.
Addressing this problem requires performing variable selection.
Three steps were taken to do so.
First, variables and categories that did not occur within one subset of the data were omitted.
Secondly, some dummy variables were redundant because some studies had identical values on multiple dummy variables.
As such variables are identical, their effects cannot be distinguished.
Only one of these redundant dummy variables was retained, and its name was updated to reflect all redundant dummies it represents.
Thirdly, despite these measures, many meta-regression models dropped all or some of the predictors,
or failed to converge entirely, suggesting the models were empirically non-identified.
Although these models are reported below,
we advise against their substantive interpretation.

The problems with meta-regression suggests that a technique is required that performs variable selection during analysis.
Such a technique was recently developed: Bayesian penalized meta-regression (BRMA), as implemented in the `pema` R-package (Van Erp S. (2021)).
By imposing a regularizing (horseshoe) prior on the regression coefficients,
BRMA shrinks all coefficients towards zero, which aids empirical model identification.
Coefficients must overwhelm the prior in order to become significantly different from zero.
Thus, this method also performs variable selection: identifying which moderators are important in predicting the effect size.
The resulting regression coefficients are negatively biased by design, but the estimate of residual heterogeneity τ2τ2  is unbiased.
Note that, as this is a Bayesian model, inference is based on credible intervals.
A credible interval is interpreted as follows: The population value falls within this interval with 95% probability (certainty).
This is different from the interpretation of frequentist confidence intervals, which are interpreted as follows: In the long run, 95% of confidence intervals contain the population value.

To examine the effect of a categorical variable,
a reference category must be chosen.
Dummy variables encode the difference between each remaining category and this reference category.
When examining the results,
the intercept represents the expected effect size for a study that falls within the reference category for all categorical variables.
The effect of dummy variables represents the difference of that category with the reference category.
If a dummy variable has a significant effect, that means that that group’s mean differs significantly from the reference category’s mean (i.e., from the intercept).

Note that in penalized regression, predictors are usually standardized.
However, the effect of standardized dummies cannot be meaningfully interpreted.
Therefore, only continuous predictors were standardized in this analysis.
This may give dummy variables a slight advantage, leading them to become significant sooner than continuous ones.

```
# Refcats now provided in different format
refcats <- read.csv("../reference_categories.csv", stringsAsFactors = FALSE)
refcats <- lapply(samples, function(s){
    out <- refcats[refcats$sample == s, -1]
    data.frame(Variable = names(out),
                      Reference = unlist(out))
})
names(refcats) <- samples
modres <- lapply(samples, function(p){
  df <- dat[dat$Sample == p, ]
  mods_cont <- mods[!mods %in% cat]
  
  constcols <- sapply(df[mods], function(i){length(unique(i)) < 2})
  constcolnames <- names(constcols)[constcols]
  mods <- mods[!mods %in% constcolnames]
  mods <- mods[mods %in% names(df)]
  dfmod <- df[, mods, drop = FALSE]
  
  # Find ref cat
  # Changed, because reference categories are now provided by researchers
  catvars <- sapply(dfmod, class) == "factor"
  catvars <- names(catvars)[catvars]
  refcat <- as.data.frame(refcats[[p]])
  dfmod[catvars] <- lapply(catvars, function(c){
    v <- dfmod[[c]]
    relevel(v, ref = refcat$Reference[match(tolower(c), refcat$Variable)])
  })
  completecases <- complete.cases(dfmod)
  modmat <- model.matrix(~., dfmod)
  
  constcols <- colSums(modmat) == 0 | colSums(modmat) == nrow(modmat)
  modmat <- modmat[, !constcols, drop = FALSE]
  modmatlist <- as.list(data.frame(modmat))
  dupcols <- duplicated(modmatlist)

    modmat <- modmat[, !dupcols, drop = FALSE]
    these_dups <- modmatlist[dupcols]
    modmatlist <- as.list(data.frame(modmat))
    for(i in 1:length(these_dups)){
      idnum <- which(duplicated(c(modmatlist, these_dups[i]), fromLast = T))
      colnames(modmat)[idnum] <- paste0(colnames(modmat)[idnum], ";", names(these_dups)[i])
    }

  
  yi_notcentered <- df$yi[completecases]
  res <- try(rma.mv(yi = yi_notcentered, V = df$vi[completecases], random = list(~ 1 | id_experiment, ~ 1 | id_es), data = df[completecases,], intercept = TRUE, mods = modmat))
  if(inherits(res, "try-error")){
    tabres <- NULL
  } else {
      vifs <- metafor::vif(res)
    confints <- confint(res)
  tabres <- data.frame(Parameter = c(rownames(res$b), "Tau2b", "Tau2w"),
                       Estimate = c(res$b, res$sigma2),
                       ci.lb = c(res$ci.lb, confints[[1]]$random[1,2], confints[[2]]$random[1,2]),
                       ci.ub = c(res$ci.ub, confints[[1]]$random[1,3], confints[[2]]$random[1,3]),
                       p = c(res$pval, NA, NA),
                       sig = c(c("", "*")[(res$pval < .05)+1], c("", "*")[(c(confints[[1]]$random[1,2], confints[[2]]$random[1,2])> 1e-4)+1]),
                       VIF = c(NA, vifs$vifs, NA, NA))
  tabres$CI <- conf_int(lb = tabres$ci.lb, ub = tabres$ci.ub)
  tabres[c("ci.lb", "ci.ub")] <- NULL
  tabres$Sample <- p
  write.csv(tabres, paste0("rma_mods_", p, ".csv"))
  }
  
  moddat <- data.frame(df[completecases, c("yi", "vi")], modmat)
  df_cats <- sapply(moddat, function(x){all(x %in% c(0, 1))})
  std <- moddat[, !df_cats, drop = FALSE]
  std <- scale(std[, -c(1:2), drop = FALSE])
  df_cats <- moddat[, df_cats, drop = FALSE]
  
  standardize <- list(
    center = c(attr(std,"scaled:center"), rep(0, ncol(df_cats))),
    scale = c(attr(std,"scaled:scale"), rep(1, ncol(df_cats)))
  )
  moddat <- cbind(yi = yi_notcentered, vi = df$vi[completecases], std, df_cats)
  moddat$study <- df$id_experiment[completecases]
  pma <- brma(yi ~., study = "study", data = moddat, standardize = standardize, iter = 10000)
  saveRDS(pma, paste0("pma_", p, ".RData"))
  sumpma <- data.frame(summary(pma)$coefficients)
  sumpma <- cbind(rownames(sumpma), sumpma)
  names(sumpma)[1:3] <- c("Parameter", "Estimate", "se")
  sumpma <- sumpma[!sumpma$Parameter == "tau", ]
  rownames(sumpma) <- NULL
  sumpma$CI <- conf_int(lb = sumpma$X2.5., ub = sumpma$X97.5.)
  pstars <- c("*", "")[as.integer(apply(sumpma[, c("X2.5.", 
                                                   "X97.5.")], 1, function(x) {
                                                     sum(sign(x)) == 0
                                                   })) + 1]
  sumpma$p <- pstars
  sumpma[c("sd", grep("^X", names(sumpma), value = T))] <- NULL
  write.csv(sumpma, paste0("brma_mods_", p, ".csv"))
  
  list(rma = tabres,
       brma = sumpma,
       refcat = refcat)
})
names(modres) <- samples
saveRDS(modres, "modres.RData")
```

### 3.5.1 Classic meta-regression

```
modres <- readRDS("modres.RData")
```

Note that analyses containing VIF values greater than 5 should be regarded as problematic, due to multicolinearity.
This applies to nearly all models.

```
  p <- samples[1]
tb <- modres[[1]]$refcat
    # knitr::kable(tb, caption = paste0("Reference category for each variable for process ", p))
    datatable(
          tb,
          rownames = FALSE,
          caption = paste0("Reference category for each variable for sample ", p))
```

Show 102550100 entries

Search:

Reference category for each variable for sample EPM

| Variable | Reference |
| --- | --- |
| ssri | Fluoxetine |
| frequency | Chronic |
| disease | Stress |
| species | Rat |
| sex | Male |
| pretested | No |
| sensitivity | None |
| usv\_test\_type |  |
| sih\_test\_type |  |

Showing 1 to 9 of 9 entries

Previous1Next

```
if(!is.null(modres[[1]][["rma"]])){
  p <- samples[1]
    tb <- modres[[1]]$rma
    tb$format <- as.integer(tb$p < .05)
    formatStyle(
      formatRound(
        datatable(
          tb,
          rownames = FALSE,
          caption = paste0("Meta-regression coefficients for ", p),
          options = list(columnDefs = list(list(
            targets = 6, visible = FALSE
          )),
          "pageLength" = nrow(tb))
        ),
        columns = c('Estimate', 'p', "VIF"),
        digits = 2
      ),
      "format",
      target = "row",
      backgroundColor = styleEqual(c(0, 1), c('gray', 'white'))
    )
} else {
  cat("Model for ", samples[1], " did not converge.")
}
```

Show 10192550100 entries

Search:

Meta-regression coefficients for EPM

| Parameter | Estimate | p | sig | VIF | CI | format |
| --- | --- | --- | --- | --- | --- | --- |
| intrcpt | -0.72 | 0.00 | \* |  | [-1.16, -0.29] | 1 |
| ssriCitalopram | 0.07 | 0.82 |  | 1.34 | [-0.51, 0.64] | 0 |
| ssriEscitalopram | 0.54 | 0.08 |  | 1.24 | [-0.06, 1.15] | 0 |
| ssriFluvoxamine | -0.02 | 0.94 |  | 1.12 | [-0.54, 0.50] | 0 |
| ssriParoxetine | 0.19 | 0.33 |  | 1.25 | [-0.19, 0.57] | 0 |
| ssriSertraline | 0.44 | 0.16 |  | 1.12 | [-0.17, 1.05] | 0 |
| frequencyAcute | 0.35 | 0.04 | \* | 1.27 | [0.02, 0.67] | 1 |
| frequencySubchronic | 0.67 | 0.10 |  | 1.38 | [-0.13, 1.47] | 0 |
| diseaseHealthy | 0.50 | 0.00 | \* | 1.42 | [0.19, 0.82] | 1 |
| diseaseOther | 0.18 | 0.55 |  | 1.25 | [-0.41, 0.78] | 0 |
| speciesMouse | -0.29 | 0.13 |  | 1.14 | [-0.68, 0.09] | 0 |
| speciesOther | -0.25 | 0.79 |  | 1.09 | [-2.05, 1.56] | 0 |
| sexBoth | -0.92 | 0.01 | \* | 1.05 | [-1.57, -0.27] | 1 |
| sexFemale | -0.42 | 0.36 |  | 1.19 | [-1.32, 0.48] | 0 |
| sexNR | -0.86 | 0.14 |  | 1.35 | [-2.02, 0.30] | 0 |
| hed | -0.08 | 0.25 |  | 1.16 | [-0.21, 0.06] | 0 |
| pretested | 0.70 | 0.38 |  | 1.08 | [-0.87, 2.27] | 0 |
| Tau2b | 0.50 |  | \* |  | [0.29, 0.81] |  |
| Tau2w | 0.15 |  | \* |  | [0.06, 0.29] |  |

Showing 1 to 19 of 19 entries

Previous1Next

```
  p <- samples[2]
tb <- modres[[2]]$refcat
    # knitr::kable(tb, caption = paste0("Reference category for each variable for process ", p))
    datatable(
          tb,
          rownames = FALSE,
          caption = paste0("Reference category for each variable for sample ", p))
```

Show 102550100 entries

Search:

Reference category for each variable for sample MB

| Variable | Reference |
| --- | --- |
| ssri | Fluoxetine |
| frequency | Chronic |
| disease | Stress |
| species | Mouse |
| sex | Male |
| pretested | No |
| sensitivity |  |
| usv\_test\_type |  |
| sih\_test\_type |  |

Showing 1 to 9 of 9 entries

Previous1Next

```
if(!is.null(modres[[2]][["rma"]])){
  p <- samples[2]
    tb <- modres[[2]]$rma
    tb$format <- as.integer(tb$p < .05)
    formatStyle(
      formatRound(
        datatable(
          tb,
          rownames = FALSE,
          caption = paste0("Meta-regression coefficients for ", p),
          options = list(columnDefs = list(list(
            targets = 6, visible = FALSE
          )),
          "pageLength" = nrow(tb))
        ),
        columns = c('Estimate', 'p', "VIF"),
        digits = 2
      ),
      "format",
      target = "row",
      backgroundColor = styleEqual(c(0, 1), c('gray', 'white'))
    )
} else {
  cat("Model for ", samples[2], " did not converge.")
}
```

Show 10172550100 entries

Search:

Meta-regression coefficients for MB

| Parameter | Estimate | p | sig | VIF | CI | format |
| --- | --- | --- | --- | --- | --- | --- |
| intrcpt | -0.58 | 0.61 |  |  | [-2.81, 1.65] | 0 |
| ssriCitalopram | -0.16 | 0.59 |  | 1.31 | [-0.74, 0.42] | 0 |
| ssriEscitalopram | 0.04 | 0.97 |  | 1.40 | [-1.73, 1.80] | 0 |
| ssriFluvoxamine | 0.49 | 0.10 |  | 1.34 | [-0.10, 1.08] | 0 |
| ssriParoxetine | -0.29 | 0.41 |  | 1.26 | [-0.96, 0.39] | 0 |
| ssriSertraline | -1.72 | 0.19 |  | 1.02 | [-4.28, 0.85] | 0 |
| frequencyAcute | -0.92 | 0.11 |  | 2.22 | [-2.04, 0.20] | 0 |
| frequencySubchronic | -0.39 | 0.64 |  | 2.07 | [-2.03, 1.24] | 0 |
| diseaseHealthy | -0.06 | 0.96 |  | 4.07 | [-2.38, 2.25] | 0 |
| diseaseOther | -0.42 | 0.75 |  | 3.81 | [-3.02, 2.18] | 0 |
| sexBoth | 0.86 | 0.17 |  | 1.33 | [-0.37, 2.09] | 0 |
| sexFemale | 0.58 | 0.40 |  | 1.21 | [-0.78, 1.93] | 0 |
| sexNR | -3.14 | 0.06 |  | 1.01 | [-6.46, 0.18] | 0 |
| hed | -0.39 | 0.00 | \* | 1.10 | [-0.52, -0.26] | 1 |
| pretested | 0.58 | 0.24 |  | 1.12 | [-0.39, 1.55] | 0 |
| Tau2b | 0.46 |  | \* |  | [0.15, 1.00] |  |
| Tau2w | 0.71 |  | \* |  | [0.43, 1.11] |  |

Showing 1 to 17 of 17 entries

Previous1Next

```
  p <- samples[3]
tb <- modres[[3]]$refcat
    # knitr::kable(tb, caption = paste0("Reference category for each variable for process ", p))
    datatable(
          tb,
          rownames = FALSE,
          caption = paste0("Reference category for each variable for sample ", p))
```

Show 102550100 entries

Search:

Reference category for each variable for sample SIH

| Variable | Reference |
| --- | --- |
| ssri | Fluoxetine |
| frequency | Chronic |
| disease | Stress |
| species | Mouse |
| sex | Male |
| pretested | No |
| sensitivity |  |
| usv\_test\_type |  |
| sih\_test\_type | Individual |

Showing 1 to 9 of 9 entries

Previous1Next

```
if(!is.null(modres[[3]][["rma"]])){
  p <- samples[3]
    tb <- modres[[3]]$rma
    tb$format <- as.integer(tb$p < .05)
    formatStyle(
      formatRound(
        datatable(
          tb,
          rownames = FALSE,
          caption = paste0("Meta-regression coefficients for ", p),
          options = list(columnDefs = list(list(
            targets = 6, visible = FALSE
          )),
          "pageLength" = nrow(tb))
        ),
        columns = c('Estimate', 'p', "VIF"),
        digits = 2
      ),
      "format",
      target = "row",
      backgroundColor = styleEqual(c(0, 1), c('gray', 'white'))
    )
} else {
  cat("Model for ", samples[3], " did not converge.")
}
```

Show 102550100 entries

Search:

Meta-regression coefficients for SIH

| Parameter | Estimate | p | sig | VIF | CI | format |
| --- | --- | --- | --- | --- | --- | --- |
| intrcpt | -0.10 | 0.90 |  |  | [-1.69, 1.48] | 0 |
| ssriEscitalopram | 0.01 | 0.99 |  | 4.36 | [-1.96, 1.99] | 0 |
| ssriFluvoxamine | -2.35 | 0.00 | \* | 1.57 | [-3.39, -1.32] | 1 |
| ssriParoxetine | -0.52 | 0.37 |  | 3.35 | [-1.67, 0.62] | 0 |
| frequencyAcute | 0.46 | 0.47 |  | 2.82 | [-0.79, 1.70] | 0 |
| speciesRat | -0.61 | 0.46 |  | 1.80 | [-2.23, 1.00] | 0 |
| hed | -0.25 | 0.46 |  | 2.06 | [-0.92, 0.41] | 0 |
| sih\_test\_type | -0.21 | 0.74 |  | 4.29 | [-1.44, 1.03] | 0 |
| Tau2b | 0.00 |  |  |  | [0.00, 10.00] |  |
| Tau2w | 0.00 |  |  |  | [0.00, 0.58] |  |

Showing 1 to 10 of 10 entries

Previous1Next

```
  p <- samples[4]
tb <- modres[[4]]$refcat
    # knitr::kable(tb, caption = paste0("Reference category for each variable for process ", p))
    datatable(
          tb,
          rownames = FALSE,
          caption = paste0("Reference category for each variable for sample ", p))
```

Show 102550100 entries

Search:

Reference category for each variable for sample USV

| Variable | Reference |
| --- | --- |
| ssri | Fluoxetine |
| frequency | Chronic |
| disease | Healthy |
| species | Rat |
| sex | Both |
| pretested | No |
| sensitivity |  |
| usv\_test\_type | Separation induced |
| sih\_test\_type |  |

Showing 1 to 9 of 9 entries

Previous1Next

```
if(!is.null(modres[[4]][["rma"]])){
  p <- samples[4]
    tb <- modres[[4]]$rma
    tb$format <- as.integer(tb$p < .05)
    formatStyle(
      formatRound(
        datatable(
          tb,
          rownames = FALSE,
          caption = paste0("Meta-regression coefficients for ", p),
          options = list(columnDefs = list(list(
            targets = 6, visible = FALSE
          )),
          "pageLength" = nrow(tb))
        ),
        columns = c('Estimate', 'p', "VIF"),
        digits = 2
      ),
      "format",
      target = "row",
      backgroundColor = styleEqual(c(0, 1), c('gray', 'white'))
    )
} else {
  cat("Model for ", samples[4], " did not converge.")
}
```

Show 10172550100 entries

Search:

Meta-regression coefficients for USV

| Parameter | Estimate | p | sig | VIF | CI | format |
| --- | --- | --- | --- | --- | --- | --- |
| intrcpt | -1.16 | 0.15 |  |  | [-2.72, 0.40] | 0 |
| ssriCitalopram | 0.60 | 0.08 |  | 1.08 | [-0.08, 1.27] | 0 |
| ssriEscitalopram | -1.30 | 0.00 | \* | 1.86 | [-2.09, -0.51] | 1 |
| ssriFluvoxamine | -0.35 | 0.56 |  | 1.48 | [-1.52, 0.82] | 0 |
| ssriParoxetine | -0.76 | 0.03 | \* | 2.89 | [-1.44, -0.09] | 1 |
| ssriSertraline | -0.08 | 0.84 |  | 2.04 | [-0.82, 0.67] | 0 |
| frequencyAcute | 0.39 | 0.56 |  | 1.18 | [-0.92, 1.71] | 0 |
| diseaseOther | 0.22 | 0.61 |  | 1.04 | [-0.63, 1.08] | 0 |
| speciesMouse | 0.99 | 0.43 |  | 1.38 | [-1.50, 3.49] | 0 |
| sexFemale | -1.50 | 0.45 |  | 3.16 | [-5.44, 2.43] | 0 |
| sexMale | -0.39 | 0.78 |  | 3.54 | [-3.06, 2.28] | 0 |
| sexNR | 0.67 | 0.49 |  | 1.36 | [-1.25, 2.59] | 0 |
| hed | -0.22 | 0.00 | \* | 1.66 | [-0.38, -0.07] | 1 |
| pretested | -1.15 | 0.17 |  | 1.48 | [-2.80, 0.49] | 0 |
| usv\_test\_typePhysical stress induced | 1.75 | 0.22 |  | 4.01 | [-1.06, 4.57] | 0 |
| Tau2b | 1.01 |  | \* |  | [0.13, 9.20] |  |
| Tau2w | 0.05 |  |  |  | [0.00, 0.28] |  |

Showing 1 to 17 of 17 entries

Previous1Next

### 3.5.2 Bayesian regularized meta-regression:

```
p <- samples[1]
    tb <- modres[[1]]$brma
    tb$format <- as.integer(tb$p == "*")
    formatStyle(
      formatRound(
        datatable(
          tb,
          rownames = FALSE,
          caption = paste0("Bayesian regularized meta-regression coefficients for ", p),
          options = list(columnDefs = list(list(
            targets = 7, visible = FALSE
          )),
          "pageLength" = nrow(tb))
        ),
        columns = c('Estimate', "se", "n_eff", "Rhat"),
        digits = 2
      ),
      "format",
      target = "row",
      backgroundColor = styleEqual(c(0, 1), c('gray', 'white'))
    )
```

Show 10192550100 entries

Search:

Bayesian regularized meta-regression coefficients for EPM

| Parameter | Estimate | se | n\_eff | Rhat | CI | p |
| --- | --- | --- | --- | --- | --- | --- |
| Intercept | -0.73 | 0.00 | 8,629.26 | 1.00 | [-1.09, -0.34] | \* |
| hed | -0.03 | 0.00 | 13,408.44 | 1.00 | [-0.15, 0.06] |  |
| ssriCitalopram | 0.02 | 0.00 | 14,513.84 | 1.00 | [-0.27, 0.37] |  |
| ssriEscitalopram | 0.14 | 0.00 | 8,617.00 | 1.00 | [-0.15, 0.68] |  |
| ssriFluvoxamine | -0.02 | 0.00 | 16,030.72 | 1.00 | [-0.35, 0.25] |  |
| ssriParoxetine | 0.05 | 0.00 | 13,699.96 | 1.00 | [-0.14, 0.35] |  |
| ssriSertraline | 0.10 | 0.00 | 11,304.43 | 1.00 | [-0.18, 0.61] |  |
| frequencyAcute | 0.21 | 0.00 | 7,083.56 | 1.00 | [-0.03, 0.57] |  |
| frequencySubchronic | 0.09 | 0.00 | 10,601.91 | 1.00 | [-0.24, 0.68] |  |
| diseaseHealthy | 0.49 | 0.00 | 9,102.35 | 1.00 | [0.15, 0.82] | \* |
| diseaseOther | 0.05 | 0.00 | 15,155.18 | 1.00 | [-0.25, 0.48] |  |
| speciesMouse | -0.15 | 0.00 | 6,290.19 | 1.00 | [-0.54, 0.08] |  |
| speciesOther | -0.02 | 0.00 | 14,871.47 | 1.00 | [-0.76, 0.63] |  |
| sexBoth | -0.44 | 0.00 | 5,965.95 | 1.00 | [-1.22, 0.05] |  |
| sexFemale | -0.04 | 0.00 | 14,793.84 | 1.00 | [-0.58, 0.37] |  |
| sexNR | -0.10 | 0.00 | 11,232.80 | 1.00 | [-0.87, 0.33] |  |
| pretested | 0.16 | 0.00 | 10,450.19 | 1.00 | [-0.35, 1.28] |  |
| tau2\_w | 0.16 | 0.00 | 3,656.30 | 1.00 | [0.05, 0.30] | \* |
| tau2\_b | 0.50 | 0.00 | 5,724.60 | 1.00 | [0.28, 0.81] | \* |

Showing 1 to 19 of 19 entries

Previous1Next

```
p <- samples[2]
    tb <- modres[[2]]$brma
    tb$format <- as.integer(tb$p == "*")
    formatStyle(
      formatRound(
        datatable(
          tb,
          rownames = FALSE,
          caption = paste0("Bayesian regularized meta-regression coefficients for ", p),
          options = list(columnDefs = list(list(
            targets = 7, visible = FALSE
          )),
          "pageLength" = nrow(tb))
        ),
        columns = c('Estimate', "se", "n_eff", "Rhat"),
        digits = 2
      ),
      "format",
      target = "row",
      backgroundColor = styleEqual(c(0, 1), c('gray', 'white'))
    )
```

Show 10172550100 entries

Search:

Bayesian regularized meta-regression coefficients for MB

| Parameter | Estimate | se | n\_eff | Rhat | CI | p |
| --- | --- | --- | --- | --- | --- | --- |
| Intercept | -1.09 | 0.01 | 6,359.84 | 1.00 | [-1.91, -0.01] | \* |
| hed | -0.35 | 0.00 | 5,258.92 | 1.00 | [-0.48, -0.21] | \* |
| ssriCitalopram | -0.06 | 0.00 | 10,905.19 | 1.00 | [-0.49, 0.29] |  |
| ssriEscitalopram | 0.08 | 0.00 | 12,943.79 | 1.00 | [-0.69, 1.10] |  |
| ssriFluvoxamine | 0.28 | 0.00 | 5,572.85 | 1.00 | [-0.10, 0.89] |  |
| ssriParoxetine | -0.11 | 0.00 | 10,685.91 | 1.00 | [-0.67, 0.27] |  |
| ssriSertraline | -0.31 | 0.01 | 10,908.97 | 1.00 | [-2.14, 0.62] |  |
| frequencyAcute | -0.35 | 0.01 | 5,495.53 | 1.00 | [-1.26, 0.18] |  |
| frequencySubchronic | 0.06 | 0.00 | 11,851.95 | 1.00 | [-0.69, 0.96] |  |
| diseaseHealthy | -0.06 | 0.00 | 7,741.00 | 1.00 | [-0.95, 0.66] |  |
| diseaseOther | -0.03 | 0.00 | 11,646.40 | 1.00 | [-1.02, 0.79] |  |
| sexBoth | 0.24 | 0.00 | 7,830.84 | 1.00 | [-0.36, 1.29] |  |
| sexFemale | 0.16 | 0.00 | 11,213.90 | 1.00 | [-0.47, 1.16] |  |
| sexNR | -0.50 | 0.01 | 13,028.25 | 1.00 | [-3.03, 0.56] |  |
| pretested | 0.18 | 0.00 | 5,308.76 | 1.00 | [-0.31, 1.03] |  |
| tau2\_w | 0.72 | 0.00 | 3,453.55 | 1.00 | [0.43, 1.10] | \* |
| tau2\_b | 0.51 | 0.00 | 2,869.53 | 1.00 | [0.14, 1.03] | \* |

Showing 1 to 17 of 17 entries

Previous1Next

```
p <- samples[3]
    tb <- modres[[3]]$brma
    tb$format <- as.integer(tb$p == "*")
    formatStyle(
      formatRound(
        datatable(
          tb,
          rownames = FALSE,
          caption = paste0("Bayesian regularized meta-regression coefficients for ", p),
          options = list(columnDefs = list(list(
            targets = 7, visible = FALSE
          )),
          "pageLength" = nrow(tb))
        ),
        columns = c('Estimate', "se", "n_eff", "Rhat"),
        digits = 2
      ),
      "format",
      target = "row",
      backgroundColor = styleEqual(c(0, 1), c('gray', 'white'))
    )
```

Show 10112550100 entries

Search:

Bayesian regularized meta-regression coefficients for SIH

| Parameter | Estimate | se | n\_eff | Rhat | CI | p |
| --- | --- | --- | --- | --- | --- | --- |
| Intercept | -0.36 | 0.01 | 10,673.66 | 1.00 | [-1.62, 0.91] |  |
| hed | -0.10 | 0.00 | 20,013.83 | 1.00 | [-0.68, 0.32] |  |
| ssriEscitalopram | -0.01 | 0.00 | 14,763.86 | 1.00 | [-1.07, 1.08] |  |
| ssriFluvoxamine | -1.05 | 0.01 | 5,272.49 | 1.00 | [-2.92, 0.30] |  |
| ssriParoxetine | -0.00 | 0.00 | 14,889.02 | 1.00 | [-0.91, 0.93] |  |
| frequencyAcute | 0.04 | 0.00 | 13,493.40 | 1.00 | [-0.71, 0.93] |  |
| frequencySubchronic | -0.07 | 0.00 | 16,514.32 | 1.00 | [-1.09, 0.75] |  |
| speciesRat | -0.06 | 0.00 | 17,027.81 | 1.00 | [-1.19, 0.92] |  |
| sih\_test\_type | -0.12 | 0.00 | 11,108.71 | 1.00 | [-1.29, 0.63] |  |
| tau2\_w | 0.10 | 0.00 | 14,902.90 | 1.00 | [0.00, 0.61] | \* |
| tau2\_b | 0.82 | 0.02 | 8,751.79 | 1.00 | [0.00, 4.35] | \* |

Showing 1 to 11 of 11 entries

Previous1Next

```
p <- samples[4]
    tb <- modres[[4]]$brma
    tb$format <- as.integer(tb$p == "*")
    formatStyle(
      formatRound(
        datatable(
          tb,
          rownames = FALSE,
          caption = paste0("Bayesian regularized meta-regression coefficients for ", p),
          options = list(columnDefs = list(list(
            targets = 7, visible = FALSE
          )),
          "pageLength" = nrow(tb))
        ),
        columns = c('Estimate', "se", "n_eff", "Rhat"),
        digits = 2
      ),
      "format",
      target = "row",
      backgroundColor = styleEqual(c(0, 1), c('gray', 'white'))
    )
```

Show 10182550100 entries

Search:

Bayesian regularized meta-regression coefficients for USV

| Parameter | Estimate | se | n\_eff | Rhat | CI | p |
| --- | --- | --- | --- | --- | --- | --- |
| Intercept | -1.03 | 0.01 | 7,375.37 | 1.00 | [-1.92, -0.15] | \* |
| hed | -0.11 | 0.00 | 5,561.68 | 1.00 | [-0.28, 0.02] |  |
| ssriCitalopram | 0.24 | 0.00 | 9,175.72 | 1.00 | [-0.12, 0.94] |  |
| ssriEscitalopram | -0.43 | 0.01 | 4,144.06 | 1.00 | [-1.29, 0.06] |  |
| ssriFluvoxamine | 0.02 | 0.00 | 9,672.15 | 1.00 | [-0.54, 0.61] |  |
| ssriParoxetine | -0.12 | 0.00 | 3,309.93 | 1.00 | [-0.71, 0.19] |  |
| ssriSertraline | 0.08 | 0.00 | 9,638.90 | 1.00 | [-0.27, 0.64] |  |
| frequencyAcute | 0.03 | 0.00 | 8,344.71 | 1.00 | [-0.57, 0.75] |  |
| frequencySubchronic | -0.07 | 0.00 | 11,812.70 | 1.00 | [-0.88, 0.50] |  |
| diseaseOther | 0.08 | 0.00 | 14,473.08 | 1.00 | [-0.32, 0.71] |  |
| speciesMouse | 0.14 | 0.00 | 8,495.78 | 1.00 | [-0.47, 1.25] |  |
| sexFemale | -0.00 | 0.00 | 13,456.13 | 1.00 | [-0.89, 0.81] |  |
| sexMale | 0.13 | 0.00 | 7,030.50 | 1.00 | [-0.39, 1.04] |  |
| sexNR | -0.02 | 0.00 | 14,340.83 | 1.00 | [-0.72, 0.64] |  |
| pretested | -0.09 | 0.00 | 9,609.29 | 1.00 | [-0.93, 0.38] |  |
| usv\_test\_typePhysical.stress.induced | 0.25 | 0.01 | 6,471.79 | 1.00 | [-0.29, 1.33] |  |
| tau2\_w | 0.09 | 0.00 | 4,029.82 | 1.00 | [0.00, 0.31] | \* |
| tau2\_b | 0.68 | 0.01 | 8,296.19 | 1.00 | [0.09, 2.38] | \* |

Showing 1 to 18 of 18 entries

Previous1Next

R Core Team. 2021. *R: A Language and Environment for Statistical Computing*. Vienna, Austria: R Foundation for Statistical Computing. https://www.R-project.org/.

Van den Noortgate, Wim, José Antonio López-López, Fulgencio Marín-Martínez, and Julio Sánchez-Meca. 2015. “Meta-Analysis of Multiple Outcomes: A Multilevel Approach.” *Behavior Research Methods* 47 (4): 1274–94. https://doi.org/10.3758/s13428-014-0527-2.

Van Erp S., Van Lissa C. J. &. 2021. “Select Relevant Moderators Using Bayesian Regularized Meta-Regression.” *PsyArxiv*. https://doi.org/10.31234/osf.io/6phs5.

Van Lissa, Caspar J., Andreas M. Brandmaier, Loek Brinkman, Anna-Lena Lamprecht, Aaron Peikert, Marijn E. Struiksma, and Barbara Vreede. 2020. “WORCS: A Workflow for Open Reproducible Code in Science,” May. https://doi.org/10.17605/OSF.IO/ZCVBS.

Viechtbauer, Wolfgang et al. 2010. “Conducting Meta-Analyses in R with the Metafor Package.” *J Stat Softw* 36 (3): 1–48.
