# Supplementary material for: The effect of SSRIs on unconditioned anxiety: a systematic review and meta-analysis of animal studies
Source: Psychopharmacology (Berl). 2024 Jul 9;241(9):1731–55. doi: 10.1007/s00213-024-06645-2 (PMC11339141; doi:10.1007/s00213-024-06645-2)
Supplement: Supplementary file 4 — Supplementary Material 4 [file 213_2024_6645_MOESM4_ESM.pdf]

The effect of SSRIs on unconditioned anxiety: a systematic review and meta-analysis of animal studies

Psychopharmacology

Elise J Heesbeen, Tatum van Kampen, P Monika Verdouw, Caspar van Lissa, Elisabeth Y Bijlsma, Lucianne Groenink

Corresponding author: Lucianne Groenink, l.groenink@uu.nl

Supplementary file S4. Bayesian regularized meta-regression coefficients for all four unconditioned anxiety tests.

Table 1. Bayesian regularized meta-regression coefficients for elevated plus maze dataset

**Parameter**	**Estimate**	**95% CI**	**Significance**
Intercept	-0.73	[-1.09, -0.34]	*
HED	-0.03	[-0.15. 0.06]	
SSRI: Citalopram	0.02	[-0.27, 0.37]	
SSRI: Escitalopram	0.14	[-0.15, 0.68]	
SSRI: Fluvoxamine	-0.02	[-0.35, 0.25]	
SSRI: Paroxetine	0.05	[-0.14, 0.35]	
SSRI: Sertraline	0.10	[-0.18, 0.61]	
Duration of treatment: Acute	0.21	[-0.03, 0.57]	
Duration of treatment: Subchronic	0.09	[-0.24, 0.68]	
Disease induction: Healthy	0.49	[0.15, 0.82]	*
Disease induction: Other	0.05	[-0.25, 0.48]	
Species: Mouse	-0.15	[-0.54, 0.08]	
Species: Other	-0.02	[-0.76, 0.63]	
Sex: Both	-0.44	[-1.22, 0.05]	
Sex: Female	-0.04	[-0.58, 0.37]	
Sex: NR	-0.10	[-0.87, 0.33]	
Pretest: Yes	0.16	[-0.35, 1.28]	
Within-study variance (τ^2 within)	0.16	[0.05, 0.30]	*
Between-study variance (τ^2 between)	0.50	[0.28, 0.81]	*

*Note: * indicates a 95% CI that excludes zero. Reference categories are fluoxetine (SSRI), chronic (duration of treatment), stress (disease induction), rat (species), male (sex), and no (pretest). CI = confidence interval, HED = human equivalent dose, SSRI = selective serotonin reuptake inhibitor, NR = not reported*

Table 2. Bayesian regularized meta-regression coefficients for marble burying dataset

**Parameter**	**Estimate**	**95% CI**	**Significance**
Intercept	-1.09	[-1.91, -0.01]	*
HED	-0.35	[-0.48, -0.21]	*
SSRI: Citalopram	-0.06	[-0.49, 0.29]	
SSRI: Escitalopram	0.08	[-0.69, 1.10]	
SSRI: Fluvoxamine	0.28	[-0.10, 0.89]	
SSRI: Paroxetine	-0.11	[-0.67, 0.27]	
SSRI: Sertraline	-0.31	[-2.14, 0.62]	
Duration of treatment: Acute	-0.35	[-1.26, 0.18]	
Duration of treatment: Subchronic	0.06	[-0.69, 0.96]	
Disease induction: Healthy	-0.06	[-0.95, 0.66]	
Disease induction: Other	-0.03	[-1.02, 0.79]	
Sex: Both	0.24	[-0.36, 1.29]	
Sex: Female	0.16	[-0.47, 1.16]	
Sex: NR	-0.50	[-3.03, 0.56]	
Pretest: Yes	0.18	[-0.31, 1.03]	
Within-study variance (τ^2 within)	0.72	[0.43, 1.10]	*
Between-study variance (τ^2 between)	0.51	[0.14, 1.03]	*

*Note: * indicates a 95% CI that excludes zero. Reference categories are fluoxetine (SSRI), chronic (duration of treatment), stress (disease induction), mouse (species), male (sex), and no (pretest). CI = confidence interval, HED = human equivalent dose, SSRI = selective serotonin reuptake inhibitor, NR = not reported*

Table 3. Bayesian regularized meta-regression coefficients for ultrasonic vocalization dataset

**Parameter**	**Estimate**	**95% CI**	**Significance**
Intercept	-1.03	[-1.92, -0.15]	*
HED	-0.11	[-0.28, 0.02]	
SSRI: Citalopram	0.24	[-0.12, 0.94]	
SSRI: Escitalopram	-0.43	[-1.29, 0.06]	
SSRI: Fluvoxamine	0.02	[-0.54, 0.61]	
SSRI: Paroxetine	-0.12	[-0.71, 0.19]	
SSRI: Sertraline	0.08	[-0.27, 0.64]	
Duration of treatment: Acute	0.03	[-0.57, 0.75]	
Duration of treatment: Subchronic	-0.07	[-0.88, 0.50]	
Disease induction: Other	0.08	[-0.32, 0.71]	
Species: Mouse	0.14	[-0.47, 1.25]	
Sex: Female	0.00	[-0.89, 0.81]	
Sex: Male	0.13	[-0.39, 1.04]	
Sex: NR	-0.02	[-0.72, 0.64]	
Pretest: Yes	-0.09	[-0.93, 0.38]	
USV test type: Physical stress induced	0.25	[-0.29, 1.33]	
Within-study variance (τ^2 within)	0.09	[0.00, 0.31]	*
Between-study variance (τ^2 between)	0.68	[0.09, 2.38]	*

*Note: * indicates a 95% CI that excludes zero. Reference categories are fluoxetine (SSRI), chronic (duration of treatment), healthy (disease induction), rat (species), both (sex), no (pretest), and separation induced (USV test type). CI = confidence interval, HED = human equivalent dose, SSRI = selective serotonin reuptake inhibitor, NR = not reported, USV = ultrasonic vocalization*

Table 4. Bayesian regularized meta-regression coefficients for stress-induced hyperthermia dataset

**Parameter**	**Estimate**	**95% CI**	**Significance**
Intercept	-0.36	[-1.62, 0.91]	
HED	-0.10	[-0.68, 0.32]	
SSRI: Escitalopram	-0.01	[-1.07, 1.08]	
SSRI: Fluvoxamine	-1.05	[-2.92, 0.30]	
SSRI: Paroxetine	0.00	[-0.91, 0.93]	
Duration of treatment: Acute	0.04	[-0.71, 0.93]	
Duration of treatment: Subchronic	-0.07	[-1.09, 0.75]	
Species: Rat	-0.06	[-1.19, 0.92]	
SIH test type: Physical stress induced	-0.12	[-1.29, 0.63]	
Within-study variance (τ^2 within)	0.10	[0.00, 0.61]	*
Between-study variance (τ^2 between)	0.82	[0.00, 4.35]	*

*Note: * indicates a 95% CI that excludes zero. Reference categories are fluoxetine (SSRI), chronic (duration of treatment), healthy (disease induction), rat (species), both (sex), no (pretest), and separation induced (USV test type). CI = confidence interval, HED = human equivalent dose, SSRI = selective serotonin reuptake inhibitor, SIH = stress-induced hyperthermia*
