# Supplementary material for: The effect of SSRIs on unconditioned anxiety: a systematic review and meta-analysis of animal studies
Source: Psychopharmacology (Berl). 2024 Jul 9;241(9):1731–55. doi: 10.1007/s00213-024-06645-2 (PMC11339141; doi:10.1007/s00213-024-06645-2)
Supplement: Supplementary file 5 — Supplementary Material 5 [file 213_2024_6645_MOESM5_ESM.pdf]

The effect of SSRIs on unconditioned anxiety: a systematic review and meta-analysis of animal studies

Psychopharmacology

Elise J Heesbeen, Tatum van Kampen, P Monika Verdouw, Caspar van Lissa, Elisabeth Y Bijlsma, Lucianne Groenink

Corresponding author: Lucianne Groenink, l.groenink@uu.nl

Supplementary file S5. Locomotor activity in the elevated plus maze, marble burying and ultrasonic vocalization test.

Table 1. Effect of SSRIs on locomotor activity in elevated plus maze dataset

	**During EPM**[a]	**Not during EPM**[b]	**Total**
**=**	45	51	96
**<**	4	6	10
**>**	0	8	8
**Total**	49	65	114
**NR**	-	-	127

[a] The locomotor activity was measured during the elevated plus maze (EPM) test. [b] The locomotor activity was not measured during the elevated plus maze but with an open field test, an actophotometer, an actimeter, an infrared sensor, an automated photobeam system, a motility box or a rotarod test. '=' = no effect, '<' = reduced locomotor activity, '>' = increased locomotor activity, NR = not reported

Table 2. Effect of SSRIs on locomotor activity in marble burying dataset

	**During MB**[a]	**Not during MB**[b]	**Total**
**=**	45	72	117
**<**	1	12	13
**>**	0	1	1
**Total**	46	85	131
**NR**	-	-	58

[a] The locomotor activity was measured during the marble burying (MB) test. [b] The locomotor activity was not measured during the marble burying but with an open field test, an actophotometer, an automated activity counter, a spontaneous motor test, an automated light-beam system, an infrared sensor, or a rotarod test. '=' = no effect, '<' = reduced locomotor activity, '>' = increased locomotor activity, NR = not reported

Table 3. Effect of SSRIs on locomotor activity in ultrasonic vocalization dataset

	**During USV**[a]	**Not during USV**[b]	**Total**
**=**	0	9	9
**<**	0	0	0
**>**	0	2	2
**Total**	0	11	11
**NR**	-	-	41

[a] The locomotor activity was measured during the ultrasonic vocalization (USV) test. [b] The locomotor activity was not measured during the ultrasonic vocalization but with a negative geotaxis test or a horizontal bar test. '=' = no effect, '<' = reduced locomotor activity, '>' = increased locomotor activity, NR = not reported
